# Myostatin regulates energy homeostasis through autocrine- and paracrine-mediated microenvironment communication

**DOI:** 10.1172/JCI178303

**Published:** 2024-06-18

**Authors:** Hui Wang, Shanshan Guo, Huanqing Gao, Jiyang Ding, Hongyun Li, Xingyu Kong, Shuang Zhang, Muyang He, Yonghao Feng, Wei Wu, Kexin Xu, Yuxuan Chen, Hanyin Zhang, Tiemin Liu, Xingxing Kong

**Affiliations:** 1 State Key Laboratory of Genetic Engineering and School of Life Sciences, Shanghai Key Laboratory of Metabolic Remodeling and Health, Institute of Metabolism and Integrative Biology, Human Phenome Institute, Fudan University, Shanghai, China.; 2 Department of Sports Medicine and Arthroscopy Surgery, Huashan Hospital, Fudan University, Shanghai, China.; 3 Department of Endocrinology and Metabolism, Zhongshan Hospital, Fudan University, Shanghai, China.; 4 Shanghai Medical College, Fudan University, Shanghai, China.; 5 Department of Endocrinology, Jinshan Hospital, Fudan University, Shanghai, China.; 6 Department of Endocrinology and Metabolism, Huashan Hospital, Fudan University, Shanghai, China.; 7 School of Life Sciences, Inner Mongolia University, Hohhot, Inner Mongolia, China..

**Keywords:** Endocrinology, Adipose tissue

## Abstract

Myostatin (MSTN) has long been recognized as a critical regulator of muscle mass. Recently, there has been increasing interest in its role in metabolism. In our study, we specifically knocked out MSTN in brown adipose tissue (BAT) from mice (MSTN^ΔUCP1^) and found that the mice gained more weight than did controls when fed a high-fat diet, with progressive hepatosteatosis and impaired skeletal muscle activity. RNA-Seq analysis indicated signatures of mitochondrial dysfunction and inflammation in the MSTN-ablated BAT. Further studies demonstrated that Kruppel-like factor 4 (KLF4) was responsible for the metabolic phenotypes observed, whereas fibroblast growth factor 21 (FGF21) contributed to the microenvironment communication between adipocytes and macrophages induced by the loss of MSTN. Moreover, the MSTN/SMAD2/3-p38 signaling pathway mediated the expression of KLF4 and FGF21 in adipocytes. In summary, our findings suggest that brown adipocyte–derived MSTN regulated BAT thermogenesis via autocrine and paracrine effects on adipocytes or macrophages, ultimately regulating systemic energy homeostasis.

## Abstract 2

Mouse models reveal that MSTN from brown adipocytes governs thermogenesis in brown adipose tissue through KLF4/FGF21.

## Introduction

Brown adipose tissue (BAT) plays a crucial role in whole-body energy balance and fuel metabolism, mediating nonshivering thermogenesis in mammals exposed to subthermoneutral temperatures (1). The abundance of mitochondria and expression of uncoupling protein 1 (UCP1) in thermogenic adipocytes equip brown fat with a unique thermogenic capacity (2). Furthermore, BAT is now recognized as a dynamic endocrine organ, secreting adipokines, gaseous messengers, and microvesicles that can target distant tissues such as white adipose tissue (WAT), liver, pancreas, heart, and bone (3, 4). Experimental studies involving BAT transplantation and activation have demonstrated notable improvements in metabolism and cardiac protection through the release of endocrine factors such as insulin-like growth factor I, IL-6, and fibroblast growth factors (FGFs) (5). In a previous study, we demonstrated that KO of IFN regulatory factor 4 in brown fat cells could reduce the secretion of myostatin (MSTN, also known as growth differentiation factor 8), impairing the exercise capacity of mice (6). However, the role of MSTN in brown fat cells remains unclear.

MSTN belongs to the TGF-β superfamily and serves as a critical regulator of skeletal muscle mass (7). Inhibitors targeting the MSTN signaling pathway have been developed for the treatment of sarcopenia and muscular dystrophy (8). However, the response to MSTN inhibitors in terms of functional improvements has been inconsistent. Although increased muscle mass has been observed in most clinical trials, this often does not translate into clinically meaningful enhancements in strength (9, 10). However, targeting the MSTN signaling pathway consistently reduces fat mass (11–13). These observations align with findings from mouse studies, in which MSTN global-KO mice exhibit increased muscle mass, reduced fat deposition, improved insulin sensitivity, enhanced fatty acid oxidation, and resistance to obesity (14, 15). Subsequent studies in mice treated with MSTN inhibitors have further elucidated the role of MSTN in metabolic regulation (16, 17). Notably, clinical observations have increasingly associated variations in MSTN expression with metabolic conditions. For instance, elevated MSTN levels have been observed in individuals with obesity and insulin resistance, implicating it in the pathophysiology of metabolic syndrome (18). Conversely, reduced MSTN activity is linked to increased muscle mass and improved metabolic profiles, suggesting a protective role against metabolic dysfunction (8). Conversely, reduced MSTN activity is linked to increased muscle mass and improved metabolic profiles, suggesting a protective role against metabolic dysfunction (8). Additionally, MSTN deletion has been found to prevent age-related increases in adipose tissue mass and to partially improve obesity diabetes in mice (19). Furthermore, specific overexpression of MSTN in adipose tissue has been demonstrated to increase the metabolic rate and resistance to diet-induced obesity (DIO) (20). The dual role of MSTN in muscle and adipose tissue underscores its potential as a therapeutic target. Clinical studies have explored MSTN inhibitors in muscle-wasting diseases, noting improvements in muscle mass and preliminary indications of metabolic benefits. These observations raise compelling questions about the broader implications of MSTN modulation in metabolic health, particularly through its effects on adipose tissues.

Kruppel-like factor 4 (KLF4) is a member of a large family of zinc-finger proteins that are critical for various development processes, including differentiation, proliferation, and inflammation. KLF4 serves as an essential early regulator of adipogenesis by regulating C/EBPβ (21). Moreover, cells deficient in KLF4 exhibit mitochondrial dysfunction and impaired mitophagy (22). Specifically, in KLF4-null cells, there is a reduction in the expression of the mitophagy-associated protein Bnip3 and the antioxidant protein GSTα4 (22). Despite substantial contextual evidence of the role of KLF4 in development, the specific molecular mechanisms in metabolism, especially in BAT, are unclear.

FGF21, a member of the endocrine FGF subfamily, has pleiotropic effects on energy homeostasis. Emerging clinical evidence demonstrates that elevated circulating FGF21 can be used as a biomarker of metabolic diseases such as metabolic dysfunction–associated steatohepatitis (MASH) and type 2 diabetes (23, 24). Notably, several FGF21 analogs and mimetics have progressed to early phases of clinical trials involving patients with obesity, type 2 diabetes mellitus, or MASH (25). Global deletion of FGF21 in mice leads to impairments in cold-induced browning of inguinal white adipose tissue (iWAT), whereas administration of recombinant FGF21 increases browning and total energy expenditure in mice (26). Huang et al. reported that adipocyte-derived FGF21 exerts autocrine effects, inducing CCL11 production in adipocytes to promote recruitment of eosinophils, thereby stimulating M2 macrophage activity (27). However, it is currently unclear what regulates FGF21 in adipocytes.

The present study found that mice with brown adipocyte–specific deletion of MSTN exhibited diet-induced insulin resistance, glucose intolerance, and hepatosteatosis, contrary to the phenotypes of MSTN global-KO mice. Furthermore, BAT-specific KO of MSTN led to a marked reduction in browning and adaptive thermogenesis. Mechanistic studies revealed that MSTN regulated the expression of KLF4 and FGF21 via the SMAD2/3 and p38 signaling pathways in adipocytes. The decreased levels of KLF4 and FGF21 contributed to MSTN deficiency–induced mitochondrial dysfunction and inflammation, respectively. These findings provide critical insights into the function of MSTN in BAT and its potential as a modulator of metabolic health, paving the way for novel interventions targeting BAT function to ameliorate obesity and metabolic diseases.

## Results

### Mice with BAT-specific MSTN KO are prone to DIO.

**Figure 1:**
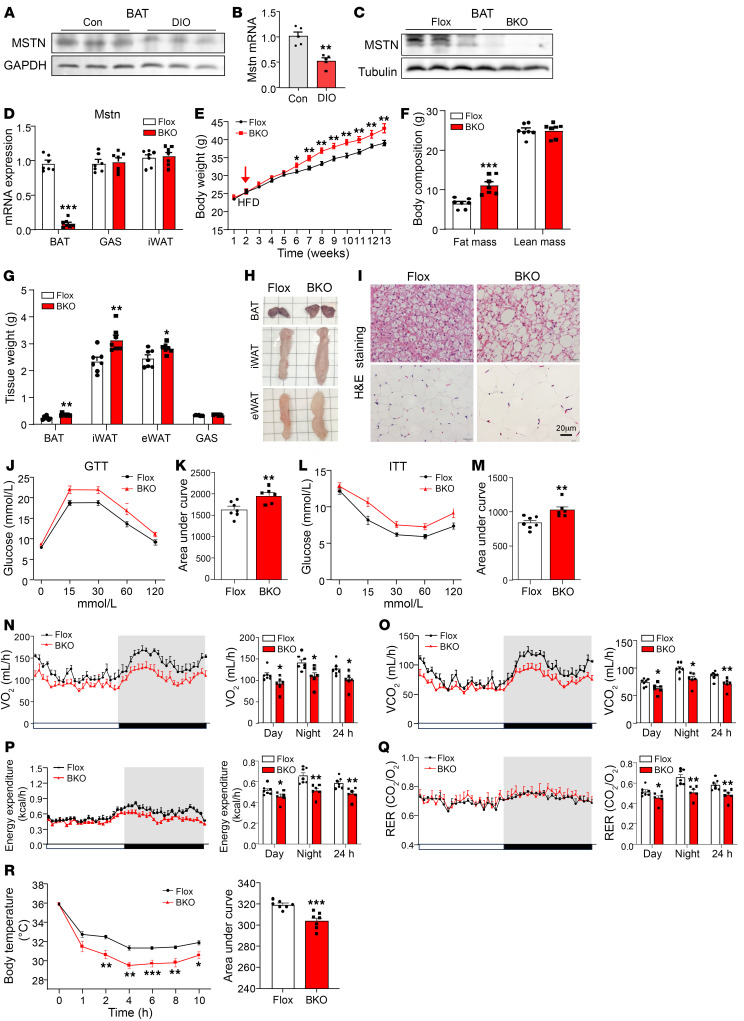
Mice with BAT-specific MSTN KO are prone to DIO. (**A**) Western blot analysis of the expression of MSTN in BAT from DIO mice (*n* = 3). (**B**) mRNA expression of *Mstn* in BAT from DIO mice (*n* = 5). Con, control. (**C**) Western blot analysis of the expression of MSTN in BAT from male BKO and Flox mice on a 12-week HFD (*n* = 3). (**D**) mRNA expression of *Mstn* in BAT, GAS, and iWAT from male BKO and Flox mice on a 12-week HFD (*n* = 7). (**E**) Body weight of male BKO and Flox mice on a HFD (*n* = 7–10). (**F**) Body composition of male BKO and Flox mice on a HFD (*n* = 7). (**G**) Weight of BAT, iWAT, epididymal WAT (eWAT), and GAS tissue from male BKO and Flox mice on a 12-week HFD (*n* = 7). (**H**) Images showing the morphology of BAT, iWAT, and eWAT. (**I**) H&E staining of BAT, iWAT of male BKO and Flox mice on a 12-week HFD. Scale bars: 20 μm. (**J**–**M**) GTTs and ITTs for male BKO and Flox mice (*n* = 6–7). (**N**–**Q**) The OCR (VO_2_), carbon dioxide production (VCO_2_), energy expenditure, and RER of male BKO and Flox mice on a 12-week HFD (*n* = 6–7). (**R**) Body temperature of male BKO and Flox mice during cold challenges (*n* = 7). All results are shown as the mean ± SEM. *P < 0.05, **P < 0.01, and ***P < 0.001, by 2-tailed Student’s *t* test.

Previous studies have suggested that BAT-derived MSTN may play a role in energy metabolism (6, 28). To further investigate the role of MSTN in regulating BAT homeostasis, we examined the expression of MSTN in response to varying nutrient states. Our findings revealed a reduction in MSTN expression in the DIO mouse model (Figure 1, A and B).

Subsequently, we generated MSTN^fl/fl^ (referred to hereafter as Flox) mice and crossed them with CAG-Cre (MSTN^ΔCAG^) mice to mimic the effects observed in MSTN global-KO mice (7). Notably, the MSTN^ΔCAG^ mice were noticeably more muscular than the control mice when fed a chow diet (Supplemental Figure 1, A and B; supplemental material available online with this article; https://doi.org/10.1172/JCI178303DS1). Additionally, the heterozygous MSTN^ΔCAG^ mice showed resistance to DIO, with reduced fat mass but increased lean mass compared with the controls (Supplemental Figure 1, C and D). Adipocyte size was also smaller in adipose tissue from heterozygous MSTN^ΔCAG^ mice on a high-fat diet (HFD) (Supplemental Figure 1E).

We then crossed the Flox mice with UCP1-Cre mice to study the thermogenic function of MSTN. The protein and mRNA levels of MSTN were markedly decreased in BAT but remained normal in other tissues (Figure 1, C and D). The protein levels of MSTN in plasma were not altered in MSTN^ΔUCP1^ mice compared with Flox mice (Supplemental Figure 2A), indicating that MSTN deletion in BAT did not affect the circulating levels of MSTN. Compared with the Flox mouse group, BAT-specific MSTN-KO male mice (referred to hereafter as MSTN^ΔUCP1^) showed no defective developmental or metabolic phenotypes in body weight and body composition when fed a normal chow (NC) diet (Supplemental Figure 2, B–I). Surprisingly, unlike the MSTN^ΔCAG^ mice, the MSTN^ΔUCP1^ mice exhibited a more pronounced increase in body weight and adiposity, without marked changes in their lean mass when fed a HFD (Figure 1, E–H). The increased body weight and adiposity were also observed in female mice (Supplemental Figure 2, J and K). To address developmental concerns, we crossed Rosa26^CAG-LSL-Cas9-tdTomato^ mice with UCP1-Cre–transgenic mice and obtained UCP1-Cre Cas9 mice. We performed in situ injection of adeno-associated virus–sgMstn (AAV-sgMstn) into BAT to specifically knock out MSTN in the BAT of UCP1-Cre mice. We found that MSTN protein levels were markedly decreased in BAT (Supplemental Figure 2L), whereas the phenotypes of AAV8-sgMstn mice were consistent with those of brown adipose tissue knockout (BKO) mice (Supplemental Figure 2, M and N). Furthermore, the MSTN^ΔUCP1^ mice displayed a more deteriorative adipose tissue phenotype characterized by larger adipocytes (Figure 1I), a finding opposite to the adipocytes observed in MSTN global-KO mice (29). Additionally, the MSTN^ΔUCP1^ mice showed insulin resistance (Figure 1, J–M). To determine whether brown adipocyte MSTN deficiency affects energy balance, we placed the mice in metabolic cages. The oxygen consumption (VO_2_), carbon dioxide production (VCO_2_), respiratory exchange rate (RER), and energy expenditure were lower in MSTN^ΔUCP1^ mice than in the control littermates (Figure 1, N–Q). To confirm the role of MSTN in adaptive thermogenesis, the mice were subjected to cold stress. As expected, MSTN^ΔUCP1^ mice were cold intolerant (Figure 1R). In summary, loss of MSTN in brown adipocytes resulted in impaired energy expenditure, which was different from the phenotypes observed among MSTN global-KO mice.

### MSTN^ΔUCP1^ mice exhibit progressive fatty liver disease.

**Figure 2:**
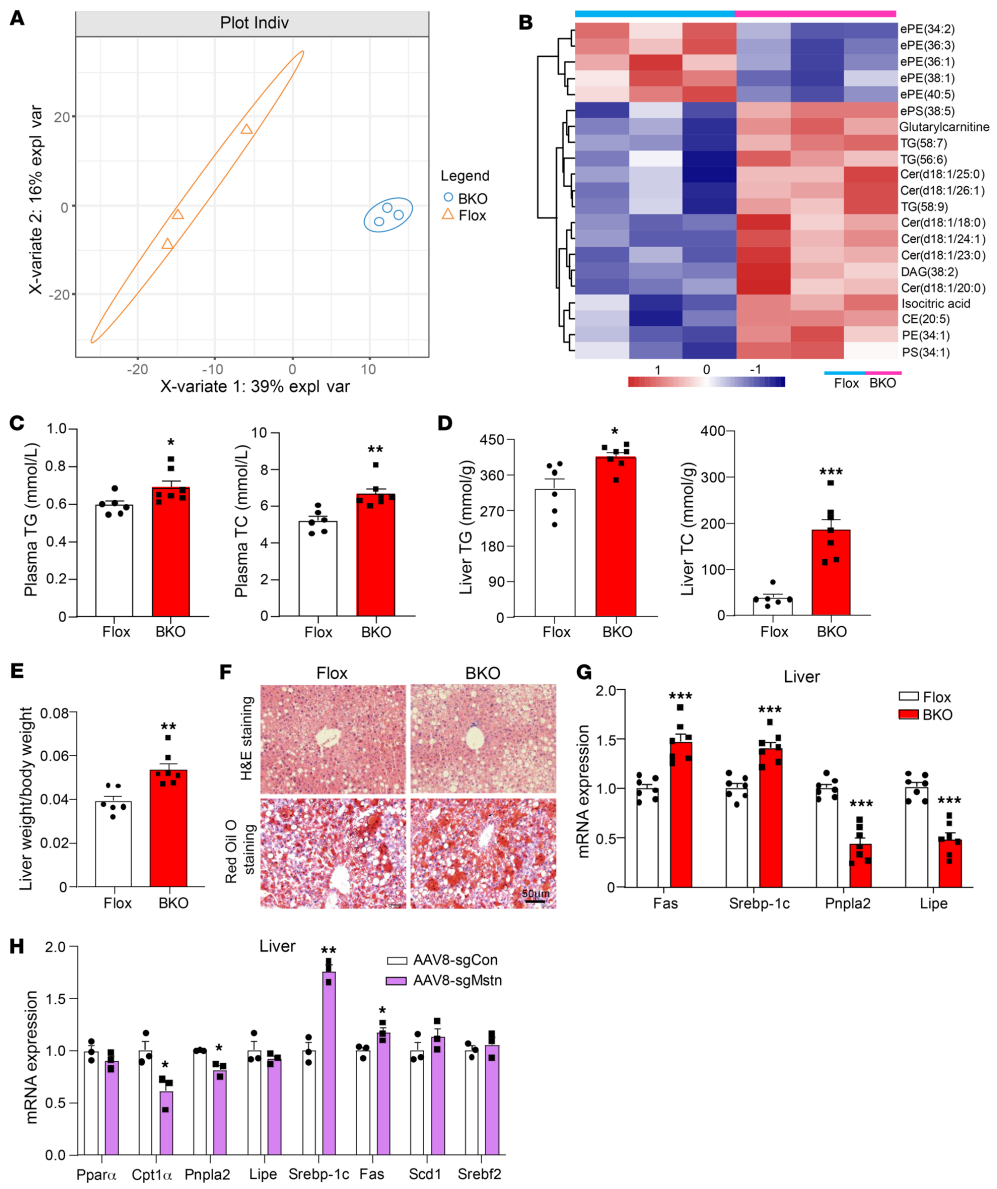
MSTN ablation in BAT shows progressive fatty liver. (**A**) PCA plot of metabolomics from the BKO and Flox groups. expl var, explained variables. (**B**) Heatmap of metabolites with significantly differential (*P* < 0.05) expression in the BKO versus the control group. (**C**) Plasma TG and TC levels in BKO and Flox mice on a 12-week HFD (*n* = 6–7). (**D**) Liver TG and TC levels in BKO and Flox mice on a 12-week HFD (*n* = 6–7). (**E**) Liver weight/body weight ratio in BKO and Flox mice on a 12-week HFD (*n* = 6–7). (**F**) H&E and Oil Red O staining of liver from BKO and Flox mice on a 12-week HFD. Scale bars: 50 μm. (**G**) Relative mRNA expression of lipid metabolism–related genes in the liver of BKO and Flox mice on a 12-week HFD (*n* = 7). (**H**) Relative mRNA expression of lipid metabolism–related genes in the liver of AAV8-sgCon and AAV8-sgMstn mice on a 12-week HFD (*n* = 3). All results are shown as the mean ± SEM. *P < 0.05, **P < 0.01, and ***P < 0.001, by 2-tailed Student’s *t* test.

To evaluate whether BAT MSTN affects systemic metabolism, we performed targeted metabolomics, encompassing 600 metabolites. Principal component analysis (PCA) revealed a clear distinction between the BKO and Flox groups (Figure 2A). Elevated levels of triglycerides (TGs) and ceramides were detected in plasma from MSTN^ΔUCP1^ mice (Figure 2B). In addition to TG levels, total cholesterol (TC) was also increased in MSTN-KO mice (Figure 2C). Given that hepatic steatosis is closely associated with obesity and insulin resistance, we next assessed the effects of MSTN deletion on hepatic lipid deposition under HFD conditions. Elevated levels of TGs and TC were observed in the livers of the MSTN^ΔUCP1^ mice (Figure 2D). The liver mass of MSTN^ΔUCP1^ mice was heavier than that of control mice after HFD feeding (Figure 2E). MSTN^ΔUCP1^ mice showed more lipid accumulation in the liver than did controls (Figure 2F). The mRNA levels of fatty acid synthesis genes, such as fatty acid synthase (*Fas*) and sterol regulatory element–binding protein 1c (*Srebp1c*) were markedly increased in livers from BKO mice compared with those from control mice (Figure 2G). Conversely, the expression of lipolysis genes, including *Pnpla2* and *Lipe*, was decreased compared with expression in controls (Figure 2G). We observed similar impairments in lipid metabolism in AAV8-sgMstn mice (Figure 2H). Thus, BAT-specific MSTN deficiency aggravated hepatic steatosis.

### Ablation of MSTN in BAT impairs skeletal muscle function.

**Figure 3:**
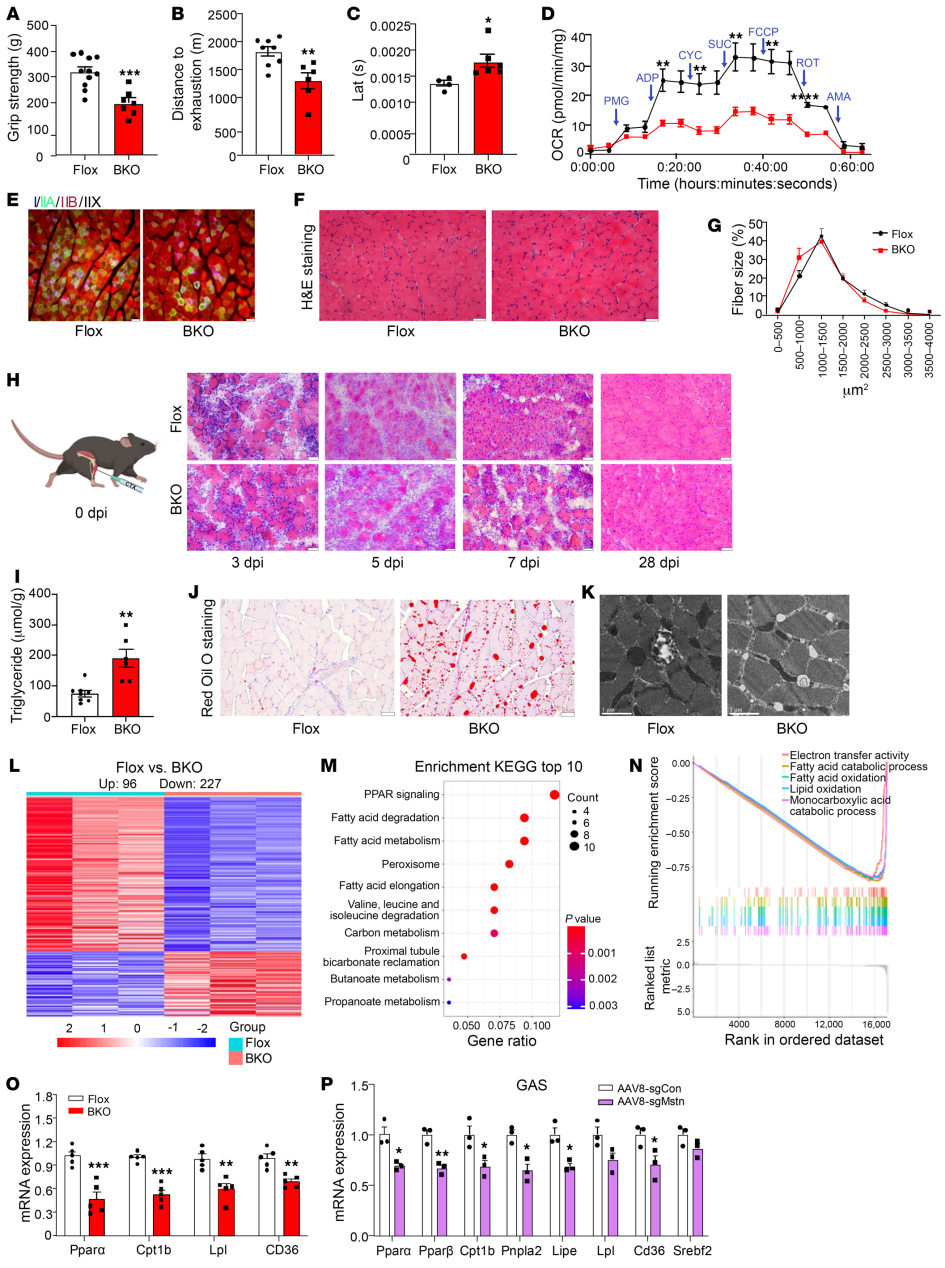
MSTN ablation in BAT impairs the function of skeletal muscle. (**A**) Grip strength of male Flox and BKO mice on a HFD (*n* = 7–11). (**B**) Total distance achieved by male Flox and BKO mice on a HFD in the exhaustion test (*n* = 6–8). (**C**) Latency (Lat) of compound muscle action potentials in GAS muscles of mice (*n* = 4–6). (**D**) OCR in GAS muscles from BKO and Flox mice on a HFD (*n* = 6). ADP, adenosine diphosphate); PMG, pyruvate, malate, glutamine; CYC, cytochrome C; SUC, succinate; FCCP, carbonyl cyanide-*p*-trifluoromethoxyphenylhydrazone; ROT, rotenone; AMA, antimycin A. (**E**) Immunofluorescence analysis of fiber type composition in GAS. The different myosin heavy chain isoforms are stained in blue (MyHC-I), green (MyHC-IIA), or red (MyHC-IIB). Scale bars: 50 μm. (**F**) Representative H&E staining of GAS from BKO and Flox mice on a HFD. Scale bars: 50 μm. (**G**) Fiber CSA distribution and median CSA of GAS. (**H**) Representative H&E staining of TA tissue, 3 days, 5 days, 7 days, and 28 days after CTX injury. Scale bars: 50 μm. (**I**) TG levels in GAS from BKO and Flox mice on a 12-week HFD (*n* = 6–8). dpi, days post injection. (**J**) Representative Oil Red O staining of GAS muscle from BKO and Flox mice on a HFD. Scale bars: 50 μm. (**K**) Representative electron micrographs of lipid droplets in muscle from male mice. Scale bars: 1 μm. (**L**) Heatmap of 323 DEGs of GAS from BKO and Flox mice on a HFD. up, upregulated; down, downregulated. (**M** and **N**) KEGG analysis and GSEA based on downregulated genes. (**O**) Relative mRNA expression of lipid metabolism–related genes in GAS of BKO and Flox mice on a 12-week HFD (*n* = 5). (**P**) Relative mRNA expression of lipid metabolism related genes in GAS of AAV8-sgCon and AAV8-sgMstn mice on a 12-week HFD (*n* = 3). All results are shown as the mean ± SEM. *P < 0.05, **P < 0.01, ***P < 0.001, and ****P < 0.0001, by 2-tailed Student’s *t* test.

Despite the comparable lean mass of MSTN^ΔUCP1^ mice compared with control mice, unlike the extremely muscular phenotype observed in MSTN global mutant animals (7), we found that skeletal muscle function was impaired. Grip strength and exercise capacity were both lower in MSTN^ΔUCP1^ mice compared with Flox mice (Figure 3, A and B). Additionally, the latency of muscle contraction was prolonged in MSTN^ΔUCP1^ mice (Figure 3C). Consistent with these findings, the oxygen consumption rate (OCR) was decreased in muscle from the MSTN^ΔUCP1^ mice (Figure 3D). Different muscle fiber types were reported to contribute to muscle strength (30). Interestingly, the MSTN^ΔUCP1^ mice exhibited a decrease in the proportion of type IIa muscle fibers, but there was no significant difference in cross-sectional area (CSA) of the fibers (Figure 3, E–G). Skeletal muscle injuries are common occurrences in daily life and exercise, and the capacity for regeneration is critical for muscle repair and functional maintenance. We injected cardiotoxin (CTX), which can induce a transient and reproducible acute injury without affecting the vasculature or nerves (31), into the tibialis anterior (TA) muscle. The MSTN^ΔUCP1^ mice exhibited delayed muscle regeneration compared with the control mice (Figure 3H).

Lipid accumulation in skeletal muscles is implicated in insulin resistance and type 2 diabetes (32). Therefore, we measured the TG levels in muscle, revealing an increase in TG levels in muscles from the MSTN^ΔUCP1^ mice (Figure 3I). The MSTN^ΔUCP1^ mice exhibited greater lipid accumulation in the gastrocnemius (GAS) than did control mice (Figure 3J). Electron microscopy images showed an elevated number of lipid droplets in muscle from MSTN^ΔUCP1^ mice compared with controls (Figure 3K). RNA-Seq analysis revealed 227 downregulated genes and 96 upregulated genes in muscle from the MSTN^ΔUCP1^ mice compared with Flox mice (Figure 3L). Pathway analysis suggested attenuation of lipid catabolism (Figure 3, M and N). Furthermore, quantitative PCR (qPCR) data revealed that expression levels of fatty acid oxidation genes were decreased in muscle from MSTN^ΔUCP1^ mice (Figure 3O). Impaired lipid metabolism was also observed in GAS muscle from AAV8-sgMstn mice, which showed lower expression levels of lipid metabolism–related genes (Figure 3P). Collectively, these findings demonstrated impaired lipid metabolism in muscle obtained from MSTN^ΔUCP1^ mice.

### Loss of MSTN attenuates mitochondrial biogenesis and mitophagy.

**Figure 4:**
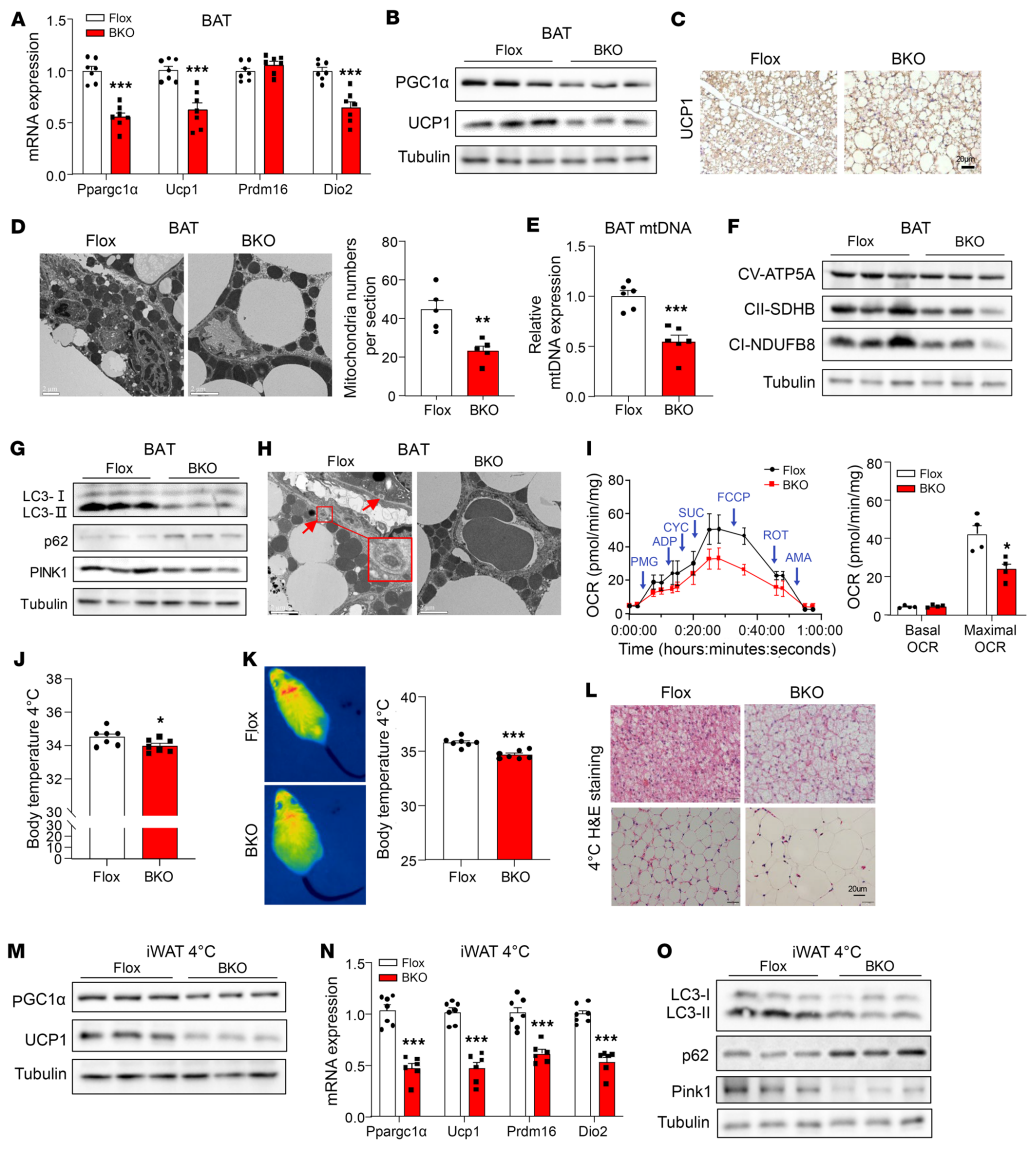
Loss of MSTN attenuates mitochondrial biogenesis and mitophagy in BAT. (**A**) Relative mRNA expression of thermogenesis-related genes in BAT of BKO and Flox mice on a 12-week HFD (*n* = 7). (**B**) Western blot analysis of PGC1-α and UCP1 in BAT (*n* = 3). (**C**) UCP1 staining of BAT from BKO and Flox mice fed a HFD for 12 weeks. Scale bars: 20 μm. (**D**) Electron microscopy images and analysis of mitochondria numbers in BAT. Scale bars: 2 μm. (**E**) Relative mtDNA expression in BAT (*n* = 6). (**F** and **G**) Western blot analysis of mitochondrial complex and mitophagy proteins (*n* = 3). (**H**) Electron microscopy images of mitophagy. Scale bars: 2 μm. (**I**) OCR of BAT and basal and maximal OCRs of mitochondrial complex II (*n* = 4). (**J**) The body temperature of BKO and Flox mice after 7 days of cold exposure (*n* = 7). (**K**) Thermography assessment of the surface temperature of the indicated mice after 7 days of cold exposure (*n* = 7). (**L**) H&E staining of BAT and iWAT from BKO and Flox mice after 7 days of cold exposure. Scale bars: 20 μm. (**M**) Western blot analysis of PGC1-α and UCP1 in iWAT from BKO and Flox mice after 7 days of cold exposure (*n* = 3). (**N**) Relative mRNA expression of thermogenesis-related genes in iWAT from mice after 7 days of cold exposure (*n* = 7). (**O**) Western blot analysis of mitophagy proteins in iWAT from BKO and Flox mice after 7 days of cold exposure (*n* = 3). All results are shown as the mean ± SEM. *P < 0.05, **P < 0.01, and ***P < 0.001, by 2-tailed Student *t* test.

MSTN has been reported to influence adipogenesis in vitro (20). To further explore this, we induced overexpression of MSTN in stromal vascular fractions (SVFs) and then induced their differentiation into adipocytes, where we observed inhibition of adipogenesis (Supplemental Figure 3A). However, we did not observe alterations in adipogenic gene expression in BAT from MSTN^ΔUCP1^ mice (Supplemental Figure 3B). Nonetheless, we noted a decrease in thermogenic gene expression in MSTN-deficient BAT (Figure 4A and Supplemental Figure 3C). Additionally, protein levels of PGC1α, a key gene involved in mitochondrial biogenesis, and uncoupling protein 1 (UCP1) were decreased as well (Figure 4, B and C). These 2 proteins were downregulated in primary brown adipocytes with knockdown of MSTN (Supplemental Figure 3D).

Given that BAT is rich in mitochondria, we analyzed mitochondrial dynamics. Electron microscopy revealed a loss of MSTN decrease in mitochondrial number in MSTN-deficient BAT compared with controls (Figure 4, D and E). Additionally, levels of some proteins associated with the mitochondrial complex, including SDHB and NDUFB8, were decreased (Figure 4F). Mitophagy, which is necessary for maintaining BAT mitochondrial integrity and optimal BAT thermogenesis, was also impaired (33). Proteins involved in mitophagy (PINK1 and LC3) were downregulated, whereas p62 was upregulated in MSTN-deficient BAT (Figure 4G) and primary brown adipocytes (Supplemental Figure 3E). This was further confirmed by electron microscopy (Figure 4H). In line with these findings, mitochondrial function was decreased, as evidenced by reduced oxygen consumption (Figure 4I).

MSTN global-KO mice have previously been shown to have induced browning of WAT (34). However, this was not the case in MSTN^ΔUCP1^ mice, which had a lower core body temperature than did control mice after 7 days of cold exposure (Figure 4J). Thermography assessment indicated a reduction in surface temperature specifically at the interscapular region in the MSTN^ΔUCP1^ mice (Figure 4K). Additionally, we noted a decreased induction of UCP1-expressing beige adipocytes and downregulation of cold-induced UCP1 expression in iWAT of MSTN^ΔUCP1^ mice (Figure 4, L–N). Furthermore, mitophagy protein levels were decreased in iWAT when MSTN^ΔUCP1^ mice were exposed to cold temperatures (Figure 4O). These findings suggested that MSTN played a critical role in adaptive thermogenesis and the browning of iWAT.

### Ablation of MSTN in BAT shows signatures of mitochondrial dysfunction and inflammation.

**Figure 5:**
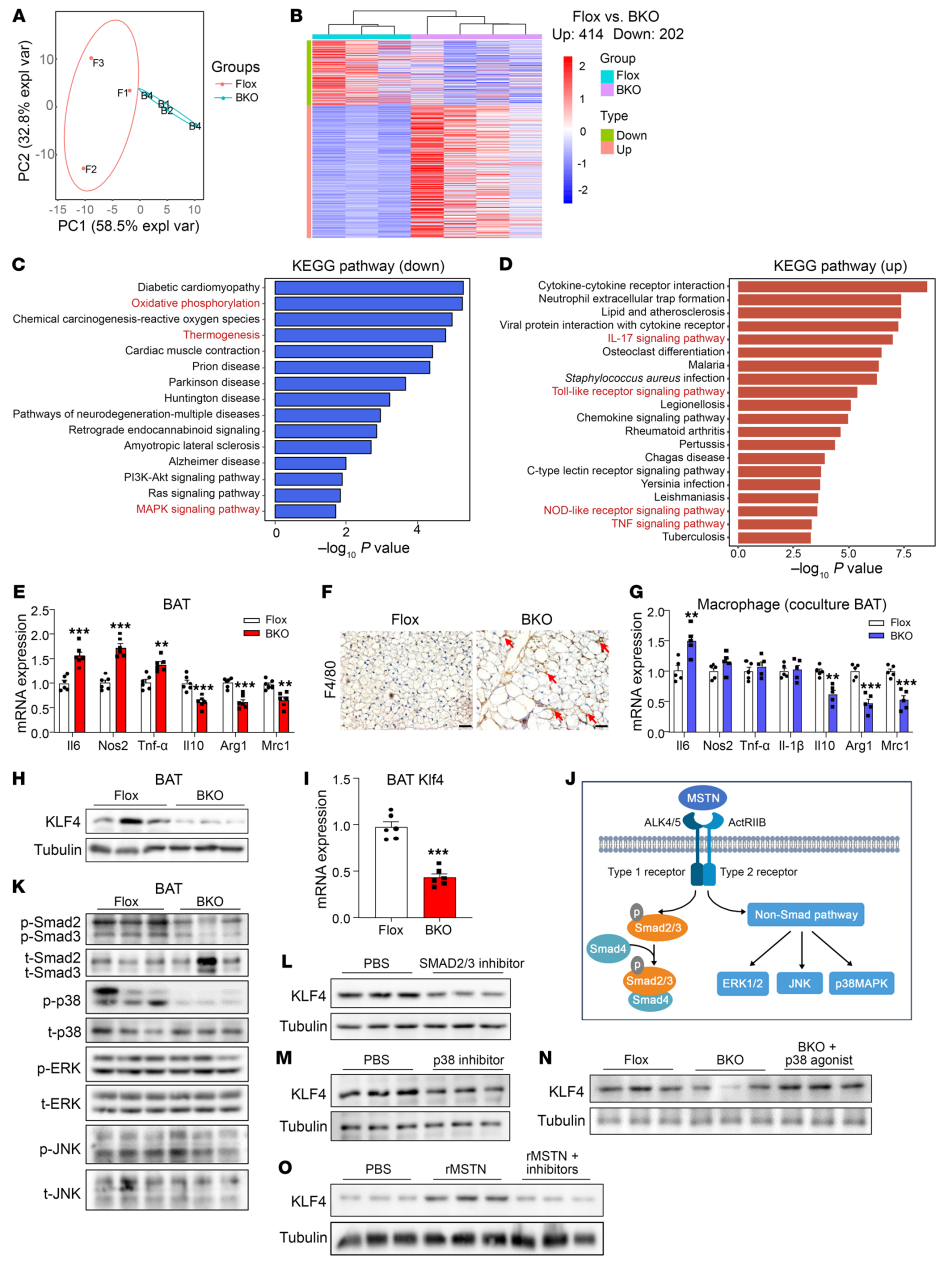
MSTN ablation in BAT shows signatures of mitochondrial dysfunction and inflammation. (**A**) PCA plot of the BAT samples from the BKO and Flox groups. (**B**) Heatmap plot comparing 414 upregulated genes and 202 downregulated genes between BKO and Flox groups. (**C**) Enriched KEGG pathways for downregulated genes. (**D**) Enriched KEGG pathways for upregulated genes. (**E**) Relative mRNA expression of inflammatory genes (*n* = 6). (**F**) F4/80 staining of BAT. Scale bars: 20 μm. (**G**) Relative mRNA expression of inflammatory genes in macrophages cocultured with BAT from BKO or Flox mice (*n* = 5). (**H**) Western blot analysis of KLF4 in BAT (*n* = 3). (**I**) Relative mRNA expression of *Klf4* in BAT (*n* = 6). (**J**) Schematic model of the downstream pathway of MSTN. (**K**) Western blot analysis of SMAD2/3 and the non-SMAD pathway in BAT from BKO and Flox mice (*n* = 3). t, total. (**L** and **M**) Western blot analysis of KLF4 in primary brown adipocytes treated with PBS and a SMAD2/3 or p38 inhibitor (*n* = 3). (**N**) Western blot analysis of KLF4 in primary brown adipocytes from Flox and BKO mice. In the BKO plus p38 agonist group, primary brown adipocytes were treated with 1 μM dehydrocorydaline (*n* = 3). (**O**) Western blot analysis of KLF4 in primary brown adipocytes treated with PBS, rMSTN, or rMSTN plus inhibitors. In the rMSTN plus inhibitors group, primary brown adipocytes were treated with a SMAD2/3 and p38 inhibitor (*n* = 3). All results are shown as the mean ± SEM. **P < 0.01 and ***P < 0.001, by 2-tailed Student *t* test.

In our subsequent investigation, we delved into elucidating the molecular mechanism of MSTN in BAT. RNA-Seq was performed, and PCA revealed differences between the BKO and Flox groups (Figure 5A). We found that 414 differentially expressed genes (DEGs) were upregulated, while 202 DEGs were downregulated (Figure 5B). The Kyoto Encyclopedia of Genes and Genomes (KEGG) pathway analysis indicated that oxidative phosphorylation was inhibited (Figure 5C), which was consistent with the observed alterations in mitochondrial function (Figure 4). However, we found that inflammation was enhanced in MSTN-KO BAT (Figure 5D). Furthermore, mRNA levels of the M1-like macrophage marker genes, which are proinflammatory, were increased, whereas those of M2-like genes, which are antiinflammatory, were decreased (Figure 5E and Supplemental Figure 4A). F4/80 staining indicated that there were more crown-like structures in MSTN-KO BAT than in the control (Figure 5F). Nonetheless, contrasting observations were noted in BAT from MSTN^ΔCAG^ mice (Supplemental Figure 4B). To explore the potential paracrine effects of MSTN-KO adipocytes on BAT-resident macrophages, we conducted coculture experiments using Transwell systems. The gene expression panels obtained from these experiments were similar to those observed in vivo (Figure 5G). Conversely, we observed no differences in the expression of inflammatory genes between macrophages cocultured with MSTN-KO adipocytes and control mature adipocytes (Supplemental Figure 4, C and D), suggesting the involvement of additional cell types in the regulatory interplay between brown adipocytes and macrophages.

The literature underscores the multifaceted role of KLF4, an essential transcriptional factor in development, in regulating both mitochondrial activity and inflammation (35–38). Therefore, we measured KLF4 expression and found that the mRNA and protein levels of KLF4 were decreased in MSTN-KO BAT (Figure 5, H and I). Similar observations were made following the knockdown of MSTN in primary adipocytes (Supplemental Figure 4E). Furthermore, ChIP-qPCR results revealed that KLF4 bound to the Pink1 promoter at –461 to –470 and –552 to –561 (Supplemental Figure 4F). Next, we investigated the mechanism underlying the regulation of KLF4 in adipocytes. Of note, MSTN can activate both SMAD or non-SMAD pathways to execute its functions (39) (Figure 5J). The phosphorylation of SMAD2/3 was decreased upon KO of MSTN (Figure 5K and Supplemental Figure 4G). Among the non-SMAD–targeted signaling pathways, only the phosphorylation of p38 was decreased, with no changes in the levels of either ERK or JNK (Figure 5K). Treatment with inhibitors targeting SMAD2/3 (TP0427736 HCl) or p38 (SB 202190) resulted in a notable decrease in the protein levels of KLF4 (Figure 5, L and M). Conversely, administration of a p38 agonist (dehydrocorydaline) effectively restored KLF4 protein levels in MSTN-KO BAT (Figure 5N). Additionally, KLF4 expression exhibited an increase in adipocytes treated with recombined MSTN (rMSTN); however, this effect was counteracted upon treatment with TP0427736 HCl and SB 202190 (Figure 5O). These findings indicate that the p38 pathway regulated KLF4 expression (40) and that MSTN regulated KLF4 via SMAD2/3 and p38 pathways.

### KLF4 is required for the metabolic phenotypes induced by MSTN ablation.

**Figure 6:**
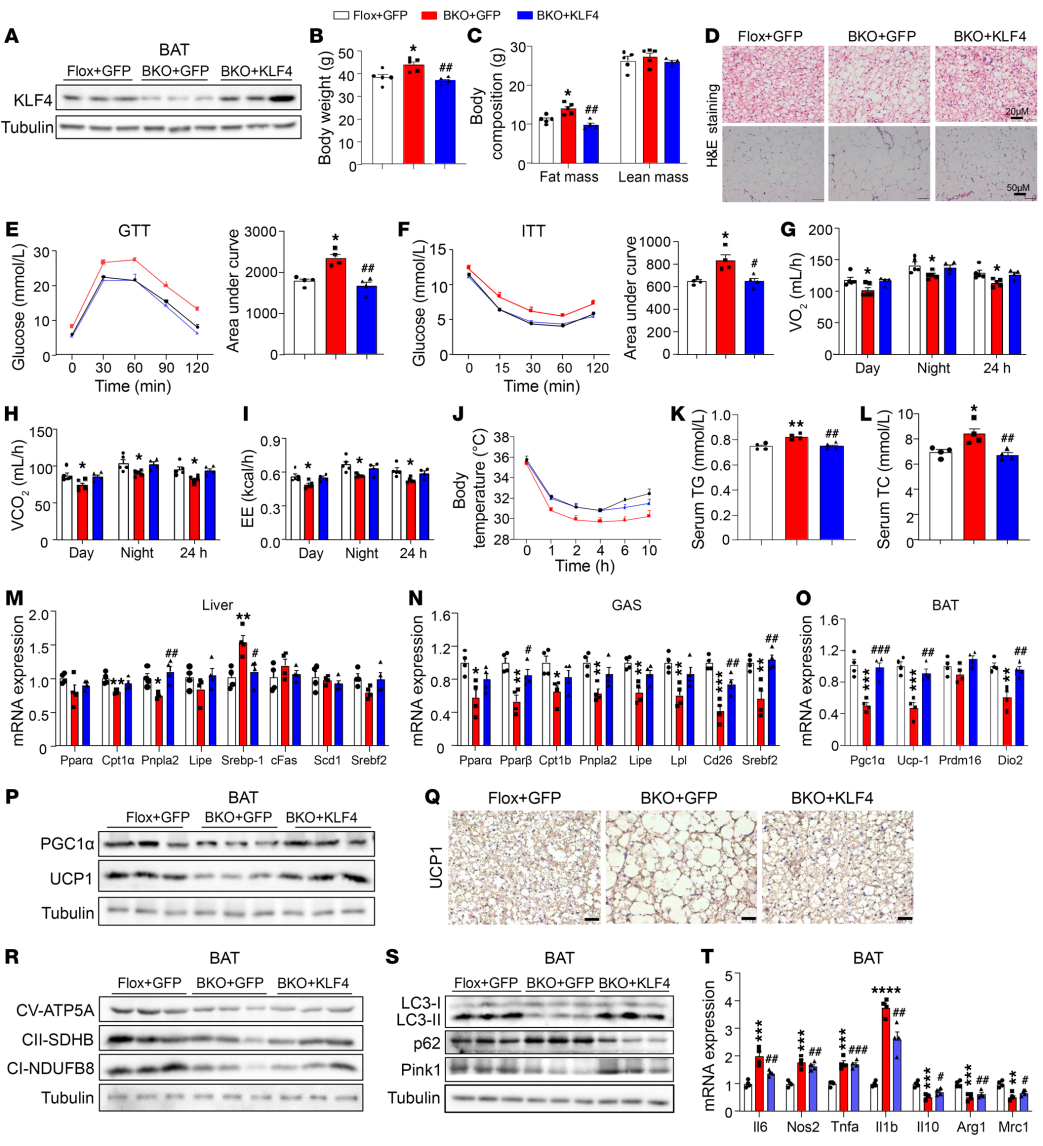
KLF4 is responsible for the metabolic phenotypes induced by MSTN ablation. (**A**) Western blot analysis of KLF4 in BAT (*n* = 3). (**B** and **C**) Body weight, fat mass, and lean mass of male Flox+GFP, BKO+GFP, and BKO+KLF4 mice on a 12-week HFD (*n* = 4–5). (**D**) H&E staining of BAT and iWAT from male Flox+GFP, BKO+GFP, and BKO+KLF4 mice on a 12-week HFD. Scale bars: 20 μm (BAT); 50 μm (iWAT). (**E** and **F**) GTT and ITT results for male Flox+GFP, BKO+GFP, and BKO+KLF4 mice on a 12-week HFD (*n* = 4). (**G**–**I**) The OCR, carbon dioxide production, and energy expenditure (EE) of male Flox+GFP, BKO+GFP, and BKO+KLF4 mice on a 12-week HFD (*n* = 4–5). (**J**) Body temperature of male Flox+GFP, BKO+GFP, and BKO+KLF4 mice during cold challenges (*n* = 4). (**K** and **L**) Plasma TG and TC levels (*n* = 4). (**M** and **N**) Relative mRNA expression of lipid metabolism–related genes in liver and GAS (*n* = 4). (**O**) Relative mRNA expression of thermogenesis-related genes in BAT (*n* = 4). (**P**) Western blot analysis of PGC1-α and UCP1 in BAT (*n* = 3). (**Q**) UCP1 staining of BAT. Scale bars: 20 μm. (**R** and **S**) Western blot analysis of mitochondrial complex and mitophagy proteins in BAT (*n* = 3). (**T**) Relative mRNA expression of inflammatory genes in BAT (*n* = 4). All results are shown as the mean ± SEM. *P < 0.05, **P < 0.01, ***P < 0.001, and ****P < 0.0001, compared with the Flox+GFP group; ^#^*P* < 0.05, ^##^*P* < 0.01, and ^###^*P* < 0.001, compared with the BKO+GFP group. A 1-way ANOVA followed by Bonferroni’s post test was used for 3-group statistical analyses.

To verify whether KLF4 is pivotal for the metabolic phenotypes induced by MSTN ablation, we induced overexpression of KLF4 in BAT via direct injection of AAV-KLF4 (Figure 6A). Notably, the expression levels of KLF4 in iWAT, GAS, and liver remained unchanged (Supplemental Figure 5, A–C). Four weeks after injection, body weight and body mass were decreased and returned to normal in AAV-KLF4–treated MSTN^ΔUCP1^ mice (Figure 6, B and C). Additionally, the adipocyte size in both BAT and iWAT was notably smaller in the AAV-KLF4–treated MSTN^ΔUCP1^ mice compared with the AAV-GFP–treated MSTN^ΔUCP1^ mice (Figure 6D). Moreover, glucose and insulin tolerance were rescued following AAV-KLF4 injection (Figure 6, E and F). Likewise, energy expenditure was rescued (Figure 6, G–I, and Supplemental Figure 5, D–G). Cold tolerance tests further demonstrated that overexpression of KLF4 ameliorated the intolerance (Figure 6J). The levels of serum TGs and TC were lower in AAV-KL4–treated MSTN^ΔUCP1^ mice than in AAV-GFP–treated MSTN^ΔUCP1^ mice (Figure 6, K and L). Additionally, the expression levels of genes related to fatty acid metabolism returned to normal in the liver and muscle (Figure 6, M and N).

The expression of thermogenic genes, such as *Pgc1a* and *Ucp1*, was decreased in MSTN-KO BAT; however, their levels were notably restored upon overexpression of KLF4 (Figure 6, O and P). UCP1 staining of BAT further revealed an increase in UCP1^+^ cells in response to KLF4 overexpression (Figure 6Q). Furthermore, overexpression of KLF4 effectively mitigated the mitochondrial dysfunction induced by the loss of MSTN, as evidenced by the restoration of proteins associated with the mitochondrial complex and mitophagy (Figure 6, R and S). In primary brown adipocytes, knockdown of MSTN decreased the protein levels of PGC1α and UCP1. However, supplementation with KLF4 successfully restored these proteins to normal levels (Supplemental Figure 5H). Although most of the MSTN-KO–induced phenotypes were counteracted by KLF4 overexpression, it is noteworthy that the expression of inflammatory genes remained unaltered (Figure 6T).

### FGF21 is responsible for the inflammatory phenotypes induced by MSTN ablation.

**Figure 7:**
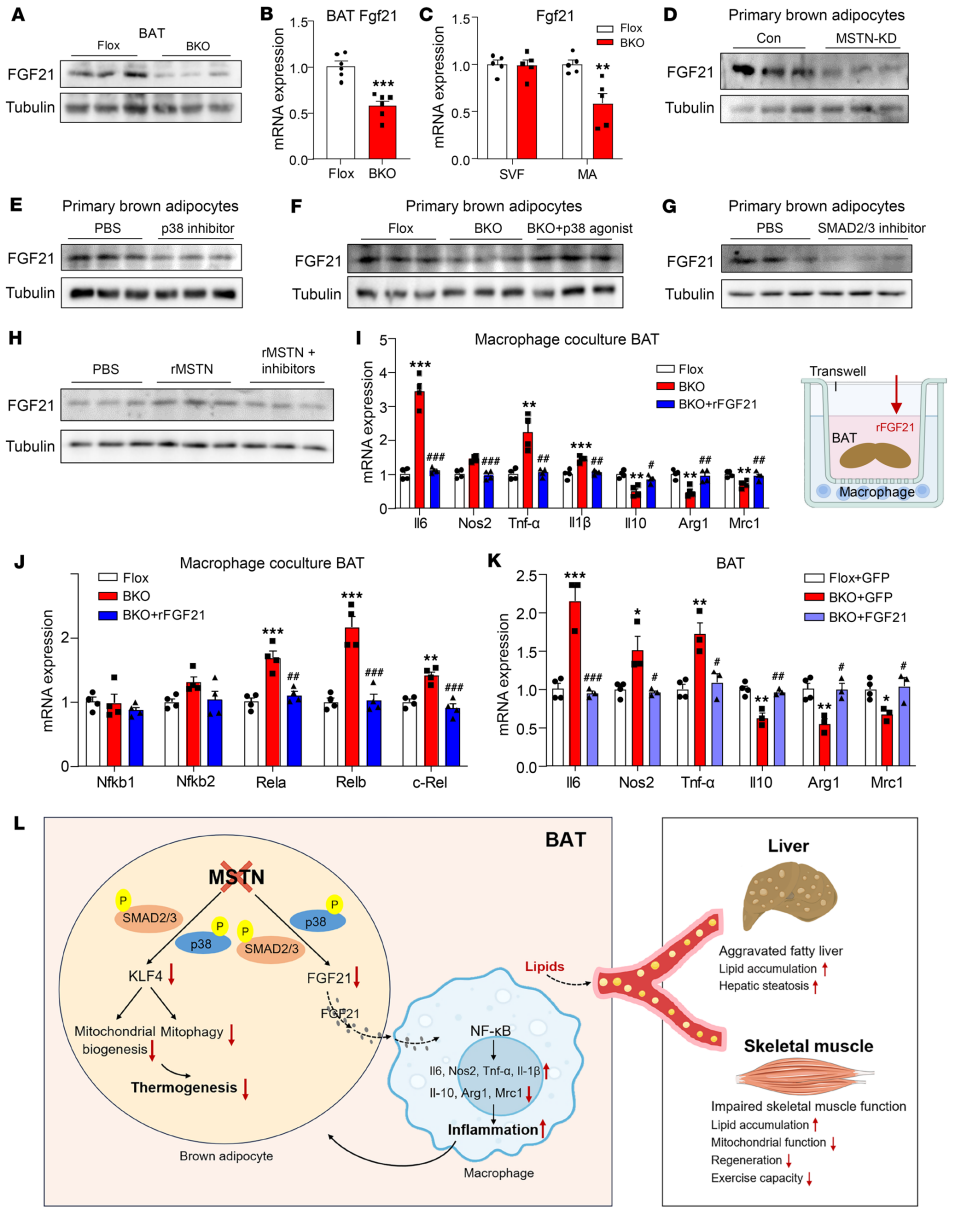
FGF21 contributes to the inflammatory phenotypes induced by MSTN ablation. (**A**) Western blot analysis of FGF21 in BAT from BKO and Flox mice on a 12-week HFD (*n* = 3). (**B**) Relative mRNA expression of *Fgf21* in BAT (*n* = 6). (**C**) Relative mRNA expression of *Fgf21* in BAT SVFs and mature adipocytes from BKO and Flox mice on a 12-week HFD (*n* = 5). (**D**) Western blot analysis of FGF21 in primary brown adipocytes of control and MSTN-knockdown groups (*n* = 3). (**E**) Western blot analysis of FGF21 in primary brown adipocytes treated with PBS or p38 inhibitor (*n* = 3). (**F**) Western blot analysis of FGF21 in primary brown adipocytes from Flox or BKO mice. In the BKO+p38 agonist group, primary brown adipocytes were treated with 1 μM dehydrocorydaline (*n* = 3). (**G**) Western blot analysis of FGF21 in primary brown adipocytes treated with a SMAD2/3 inhibitor (*n* = 3). (**H**) Western blot analysis of FGF21 in primary brown adipocytes treated with PBS, rMSTN, or rMSTN plus inhibitors (*n* = 3). In the rMSTN plus inhibitors group, primary brown adipocytes were treated with SMAD2/3 and p38 inhibitor. (**I** and **J**) Relative mRNA expression of inflammatory genes (**I**) and NF-κB signaling pathway–related genes (**J**) in macrophages cocultured with BAT from Flox and BKO mice. In the BKO+rFGF21 group, macrophages were additionally treated with 100 nM rFGF21 (*n* = 4). (**K**) Relative mRNA expression of inflammatory genes in BAT from Flox, BKO+GFP, and BKO+FGF21 mice (*n* = 3–4). (**L**) Working model. All results are shown as the mean ± SEM. **P < 0.01 and ***P < 0.001, compared with the Flox group; ^#^*P* < 0.05, ^##^*P* < 0.01, and ^###^*P* < 0.001, compared with the BKO group. A 2-tailed Student *t* test was used for 2-group statistical analyses, and 1-way ANOVA followed by Bonferroni’s post test was used for 3-group statistical analyses.

FGF21 was found to mediate the crosstalk between adipocytes and macrophages (27). We thus investigated whether FGF21 accounted for the inflammation induced by MSTN ablation. We found that the expression of FGF21 was decreased in MSTN-KO BAT (Figure 7, A and B), and the fractionation revealed that the decrease only occurred in mature adipocytes but not in SVFs (Figure 7C). Knockdown of MSTN in primary brown adipocytes also inhibited the expression of FGF21 (Figure 7D). p38 has been reported to mediate FGF21 release in mice and adipocytes (41). Primary brown adipocytes treated with a p38 inhibitor exhibited decreased FGF21 expression (Figure 7E). However, treatment with a p38 agonist restored the protein levels of FGF21 in MSTN-KO BAT (Figure 7F). Although several studies have reported that FGF21 exerts its functions via the phosphorylation levels of SMAD2/3 (42–44), inhibition of SMAD2/3 decreased the expression of FGF21 (Figure 7G). Additionally, rMSTN upregulated the expression levels of FGF21; however, the expression levels were restored to normal upon treatment with TP0427736 HCl and SB 202190 (Figure 7H).

To explore the role FGF21 in MSTN-KO–induced inflammation, we introduced recombinant FGF21 (rFGF21) into a coculture system. The addition of rFGF21 successfully reversed the expression of inflammatory genes induced by BAT from MSTN^ΔUCP1^ mice (Figure 7I). FGF21 probably inhibited macrophage-mediated inflammation by suppressing the NF-κB signaling pathway, as evidenced by the downregulation of Rela, Relb, and c-Rel in cocultured macrophages treated with rFGF21 (Figure 7J). Consistent with Xu’s study (27), our findings indicated that adipocyte-derived FGF21 may indirectly modulate macrophage-mediated inflammation, as rFGF21 had no effects on macrophages when cocultured with mature adipocytes (Supplemental Figure 6A). Additionally, a single injection of rFGF21 directly into BAT resulted in decreased expression of inflammatory genes (Supplemental Figure 6B). Importantly, this treatment did not alter serum TG or TC levels (Supplemental Figure 6, C and D). To further verify whether FGF21 is required for the MSTN-KO–induced inflammatory phenotype, we induced overexpression of FGF21 in BAT using AAV-FGF21. Interestingly, inflammation resolved in BAT of BKO mice overexpressing FGF21. However, metabolic phenotypes such as mitochondrial function, liver TGs, and lipid metabolism in the liver and GAS muscle were not restored to normal (Figure 7K and Supplemental Figure 6, E–K).

## Discussion

MSTN is well known as a key inhibitor of skeletal muscle growth. Although several studies have addressed the endocrine effect of MSTN on adipose tissue, the results have been controversial. The present study provides a series of evidence indicating that brown adipocyte–derived MSTN serves as a key triggering factor for energy homeostasis via its autocrine and paracrine actions within the metabolic niche of BAT (Figure 7L). In MSTN-KO adipocytes, the expression of KLF4 and FGF21 was decreased due to inhibition of the SMAD2/3 and p38 signaling pathways. Treatment with KLF4 ameliorated all the metabolic phenotypes induced by MSTN ablation, except inflammation. FGF21 was proven to contribute to MSTN-KO–induced inflammation. These findings demonstrate an important role of brown adipocyte–derived MSTN in metabolism, resulting in a phenotype different from those of global KO mice, suggesting that MSTN has cell- and tissue-specific effects.

Accumulating in vitro and in vivo data from diverse laboratories in recent years support the notion that inhibition of MSTN, either through pharmacological modulation or genetic inactivation, increases brown adipose characteristics, enhances energy expenditure, and provides metabolic benefits (19). However, specific inhibition of MSTN signaling, achieved through overexpression of a dominant-negative activin IIB receptor in adipocytes or skeletal muscle, reveals contrasting findings. While inhibition in muscle leads to reduced fat mass and improved insulin sensitivity, the direct effect on adipose tissue remains less pronounced, suggesting that changes in glucose metabolism and adiposity in MSTN-null mice may primarily stem from alterations in skeletal muscle function rather than direct effects on adipose tissue (45). However, Feldman et al. reported that ectopic production of MSTN, specifically in adipose tissue, induces adipose wasting (20). Consistent with this, our MSTN^ΔUCP1^ mice exhibited DIO, with insulin resistance, impaired energy expenditure, cold intolerance, and hepatosteatosis. These data indicate that the phenotypes of MSTN^ΔUCP1^ and MSTN-null mice are different. Several interpretations can be drawn. First, it is plausible that brown adipocyte–derived MSTN regulates mitochondrial function via autocrine or intracellular effects by enhancing KLF4-mediated mitochondrial turnover. Second, MSTN may regulate the expression of FGF21 in adipocytes, with FGF21 acting as a secretion factor to modulate immune cells in BAT, thereby serving as a physiological integrator of metabolism and immunity. Third, differences in adipocyte size between MSTN^ΔUCP1^ and MSTN-null mice — larger in the former and smaller in the latter — suggest distinct endocrine and paracrine roles of MSTN in adipocyte precursors. Last, variations in the inflammatory state of BAT between MSTNΔUCP1 (proinflammatory) and global-KO mice (antiinflammatory) may elicit feedback responses in adipocytes and other niche cells, potentially mediated by the role of MSTN in immune cells, particularly macrophages.

Several studies have demonstrated that mutations in the MSTN gene increase skeletal muscle mass in mice, cattle, sheep, dogs, and humans (46). However, the MSTN^ΔUCP1^ mice in this study exhibited identical lean mass with impaired exercise performances. Given that BAT thermogenesis primarily relies on fatty acids hydrolyzed from intracellular TGs (47), any impairment in BAT may result in increased TG levels. Notably, we found that serum and muscle TG levels were increased in the MSTN^ΔUCP1^ mice. Furthermore, the RNA-Seq data indicated disordered lipid metabolism pathways in the MSTN^ΔUCP1^ mice, in contrast to the global-KO mice. Additionally, plasma MSTN levels remained unchanged in the MSTN^ΔUCP1^ mice compared with the Flox mice. These data indicate that brown adipocyte–derived MSTN impairs systemic lipid metabolism.

ActRIIB serves as the type II receptor for MSTN, whereas ActRIB and TβRI act as the type I receptors (48). MSTN signaling pathways include SMAD-mediated and non-SMAD (such as p38 MAPK, JNK, ERK) pathways (39). Among these, the p38 pathway has emerged as a key regulator of BAT activation. While JNK activation is increased in adipose tissues of obese mice (49, 50), p38 activity is markedly decreased in the adipose tissue of mice with diet-induced or genetically induced (ob/ob) obesity (51). In line with this, the phosphorylation of p38 was found to be decreased in MSTN-null BAT. Our findings further demonstrate that SMAD2/3 or p38 inhibitors can mimic the effects induced by MSTN knockdown in brown adipocytes. Additionally, previous studies have indicated that the p38 signaling pathway can regulate the expression of Klf4 and Fgf21 (41, 52). In the present study, we examined a pathway for MSTN intracellular signals.

In summary, our study uncovered a physiological role of the brown adipocyte–derived MSTN/KLF4/FGF21 axis in regulating the metabolic niche in BAT, thereby regulating systemic energy homeostasis. Although both animal and clinical studies have demonstrated promising effects of MSTN antibody in reducing body weight and increasing muscle mass (8), a better understanding of MSTN in adipose tissue holds potential for novel and promising clinical applications in controlling body and fat weight, as well as animal production.

## Methods

### Sex as a biological variable

Male and female mice were used in this study.

### Animals

Mice were maintained under a 12-hour light/12-hour dark cycle at constant temperature (23°C) and humidity (50%–60%) with free access to food and water. MSTN^fl/fl^ mice were generated using CRISPR/Cas9 technology. According to the conserved region and structure of the MSTN gene, exon2-exon3 of MSTN (ENSMUSG00000026100) was set as the KO region.

#### Animal experiment design 1.

To confirm the role of MSTN in adipose tissue, we crossed MSTN^fl/fl^ (Flox) mice with CAG-Cre (MSTN^ΔCAG^) mice to mimic the effects of MSTN global KO. The male WT and MSTN^+/–^ mice were treated with a 60% HFD (PD6001, Changzhou SYSE Bio-Tec) to generate obese mice.

#### Animal experiment design 2.

To further investigate whether MSTN participates in regulating BAT homeostasis, we generated BAT-specific MSTN-KO mice. To delete MSTN expression in BAT, we crossed MSTN^fl/fl^ mice with UCP1-Cre–transgenic mice and obtained UCP1-Cre MSTN^fl/fl^ (MSTN^ΔUCP1^) mice (referred to herein as BKO mice). Cre^–^ floxed MSTN mice (i.e., MSTN^fl/fl^, referred to herein as Flox mice) were used as controls in this study. Male Flox and BKO mice were used to establish an obesity model for a 12-week 60% HFD. After the obesity modeling period, BAT, iWAT, liver, and GAS tissues were collected to examine DIO in Flox and BKO mice. To confirm the role of MSTN in adaptive thermogenesis, mice were also subjected to 1 week of 4°C cold stress to detect the adaptive thermogenesis and browning of iWAT.

#### Animal experiment design 3.

To specifically overexpress KLF4 in BAT in vivo experiments, the AAV serotype 8–encoding (AAV8-encoding) full-length KLF4 sequence (AAV-KLF4) delivery system was established. After 12-week obesity modeling, AAV-KLF4 was injected into BAT. After 3 weeks of AAV injections, BAT, iWAT, liver, and GAS samples were collected from mice to detect corresponding histological, biochemical, and molecular biological analysis.

#### Animal experiment design 4.

Rosa26^CAG-LSL-Cas9-tdTomato^ mice were crossed with UCP1-Cre–transgenic mice to obtain UCP1-Cre Rosa26^CAG-LSL-Cas9-tdTomato^ mice; male Rosa26^CAG-LSL-Cas9-tdTomato^ and male UCP1-Cre Rosa26^CAG-LSL-Cas9-tdTomato^ mice were used to establish the obesity model for a 12-week 60% HFD. Then we constructed the AAV of pAAV-U6-spgRNA (Mstn)-CMV-EGFP-WPRE, and AAV-sgMstn was injected into BAT in situ to specifically knock down MSTN in BAT. Rosa26^CAG-LSL-Cas9-tdTomato^ mice (strain no. T002249) were purchased from GemPharmatech.

#### Animal experiment design 5.

To confirm whether FGF21 contributes to the inflammation induced by MSTN ablation, we established a delivery system encoding the full-length FGF21 sequence (AAV-FGF21) of AAV type 8 (AAV8). After 12 weeks of obesity modeling, AAV-FGF21 was injected into BAT in situ to specifically induce overexpression of FGF21 in BAT. After 3 weeks of AAV injection, BAT, iWAT, liver, and GAS samples were collected from male mice for corresponding histological, biochemical, and molecular biological analyses.

#### Animal experiment design 6.

To confirm whether FGF21 contributes to the inflammation induced by MSTN ablation, we injected rFGF21 once directly into BAT of BKO mice. The dose of rFGF21 was 2 mg/kg (53). Since the half-life of rFGF21 in rodents and primates has been determined to be approximately 1–2 hours (54). After a 1-time rFGF21 injection, the BAT, iWAT, liver, and GAS samples were collected within 2 hours.

### Body composition measurement

The Minispec mq10 NMR Analyzer (Bruker) was used to measure the body composition of mice according to the manufacturer’s instructions. Briefly, mice were put in an NMR tube and loaded into the NMR machine. Body composition was measured automatically by the machine.

### Glucose and insulin tolerance tests

Glucose and insulin tolerance tests (GTTs and ITTs) were performed as previous described. Briefly, mice were fasted overnight before the GTT. Glucose (1.5 g/kg) was administered i.p., and blood glucose levels were measured 0, 15, 30, 60, and 120 minutes after injection. Mice were fasted for 6 hours before the ITT. Insulin (0.8 U/kg) was administered i.p., and blood glucose was measured 0, 15, 30, 60, and 120 minutes after injection.

### Body temperature and surface temperature

Mice were hand-restrained, and the rectal temperature was monitored using an animal digital electronic thermometer (ALC-ET03/06, Alcott Bitech). A rectal probe was gently inserted into the rectum to a depth of 2 cm. To alleviate acute stress-induced increases in body temperature, the mice were trained in advance to the measurement procedure and to the restraint every day for 3 days. An infrared thermographic camera was used to collect the surface temperature of the mice (FOTRIC 220s). For assessment of living mice, an infrared thermographic camera was placed vertically above the mouse and within less than 1 m distance from the animal. Each infrared digital image was analyzed with FOTRIC AnalyzIR software.

### Cold tolerance test

To test tolerance to cold exposure, mice were individually housed at 4°C without bedding and with free access to food and water. The core body temperature of the mice was measured using a rectal thermometer at 0, 1, 2, 4, 6, 8, and 10 hours.

### Muscle grip-strength test

In this study, the grip strength of mice was assessed using a grip-strength meter. Prior to testing, mice underwent a 2-day acclimation period with the apparatus. For the measurement, each mouse was aligned with a metal grid, where it was allowed to grip with its 4 limbs. Subsequently, a gentle tail pull was applied until the mouse voluntarily released its grip. The grip strength was determined by averaging the results from 3 consecutive tests.

### Treadmill performance test

For the treadmill test, mice underwent a 2-day acclimation period on the treadmill apparatus. The test commenced with a 5-minute warm-up phase, during which the mice were subjected to a 10° incline and a constant speed of 10 meters per minute. Following the warm-up, the treadmill speed was incrementally increased by 2 meters per minute every 5 minutes. The endpoint of the test was defined as the point of exhaustion, determined by the mouse’s inability to continue running.

### Latency measurement in the mouse sciatic nerve–GAS muscle system

Prior to the experiment, anesthesia was administered via i.p. injection of pentobarbital, with dosage adjusted according to body weight. Under sterile conditions, a unilateral surgical exposure of the sciatic nerve was performed. A precision stimulator delivered a square wave pulse of fixed duration, with stimulus intensity controlled to elicit visible muscle responses without causing tissue damage. Latency periods of the GAS muscle were recorded using needle electrodes strategically placed in the muscle to detect the onset of contraction following nerve stimulation. Data were amplified and recorded using a standard electrophysiological recording system (55).

### Preparation and delivery of cardiotoxin

A working solution of 10 μM was aliquoted into disposable Eppendorf vials. For muscle injury induction, the area was sterilized with 70% ethanol followed by injection of approximately 50 μL of the 10 μM cardiotoxin into the TA muscle belly. The injection site was carefully chosen at 1 cm below the proximal insertion of the TA, with the needle inserted at a shallow angle along the muscle belly and the syringe held at a 20° angle. In every case of cardiotoxin-induced injury, the contralateral leg was kept uninjured to serve as a control (56).

### Energy metabolism

The metabolic condition of mice that were fed a HFD for 12 weeks was determined using the TSE PhenoMaster Animal Monitoring System (TSE Systems Instruments). Body weight–matched mice were housed individually and maintained under a 12-hour light/12-hour dark cycle at 23°C with free access to food and water. The OCR, carbon dioxide production, RER, and energy expenditure were measured during the experiment. Data were analyzed using CalR, a web-based analysis tool for indirect calorimetry experiments (57). Total body mass was used as a covariate.

### Histology

BAT, iWAT, liver, and GAS tissues were fixed in a 4% paraformaldehyde solution for 24 hours and then embedded in paraffin. H&E (hematoxylin, E607317-0500; eosin, E607321-0100, Sangon Biotech) staining was performed on paraffin-embedded tissues to visualize the pattern of lipid accumulation. Oil Red O (E607319-0010, Sangon Biotech) staining was performed on OCT compound–embedded (4583, Sakura) frozen liver or GAS sections to visualize lipid droplet accumulation. UCP1 (Abcam) staining was performed to detect BAT thermogenesis, and F4/80 (Proteintech) staining was performed to detect liver lobule inflammation. Histological features were observed and captured with a light microscope (Olympus DP80, Olympus).

### Muscle fiber measurements

Each image was acquired by setting the actual dimensions using “analyze” > “set scale.” To analyze the muscle fiber CSA, we selected the “freehand selections” tool to manually outline the muscle fiber contours. Once a muscle fiber was selected, we chose “analyze” > “measure” to calculate and display the area of the selected region. These steps were repeated for all muscle fibers. The “ROI Manager” was used to manage and label multiple regions, facilitating the measurement process across multiple fibers. During the process of muscle fiber measurements, 3 mice from each group were used, and 1,000–3,000 muscle fibers per mouse were used in the calculation.

### Immunofluorescence

For the immunohistochemical analyses, 20 μm sections were prepared. Primary antibodies targeting MyHC-I (BA-D5), MyHC-IIa (SC-71), and MyHC-IIb (BF-F3) were procured from Developmental Studies Hybridoma Bank (DSHB). Secondary antibodies, namely Alexa Fluor 350 anti–mouse IgG2b (A-21140), Alexa Fluor 488 anti–mouse IgG1 (A-21121), and Alexa Fluor 555 anti–mouse IgM (A-21426), were sourced from Thermo Fisher Scientific. Imaging was conducted using a Zeiss confocal microscope, with quantitative assessments performed with Fiji software.

### Electron microscopy

BAT and GAS muscles from BKO and Flox mice fed a HFD for 12 weeks were fixed as previously described (58). Ultrathin sections of BAT and GAS muscles were prepared by ultramicrotome (Leica EM UC7), and the grids were analyzed with a JEM-2100 transmission electron microscope (HT7700, Hitachi).

### Measurement of lipids

Tissue lipids were extracted with chloroform and methanol and determined as previously described (59). TG content in the plasma, liver, and GAS tissues was measured as recommended by the manufacturer (TR0100, MilliporeSigma). Absorbance at 540 nm was measured with a microporous plate spectrophotometer (Spark).

### Muscle fiber permeabilization and mitochondrial respiration studies

GAS muscles were excised, segmented into 15–20 mg pieces, and immediately submerged in ice-cold BIOPS solution (CaK2EGTA 2.77 mM, K2EGTA 7.23 mM, Na_2_ATP 5.77 mM, MgCl_2_·6H_2_O 6.56 mM, taurine 20 mM, Na_2_ phosphocreatine 15 mM, imidazole 20 mM, DTT 0.5 mM, MES hydrate 50 mM). Fiber bundles were delicately dissected, saponin treated, and agitated in BIOPS. Subsequently, fibers were swiftly transferred to MIR05 solution (EGTA 0.5 mM, MgCl_2_·6H_2_O 3 mM, k-lactobionate 60 mM, taurine 20 mM, KH_2_PO_4_ 10 mM, HEPES 20 mM, sucrose 110 mM, BSA 1 g/L). Permeabilized fibers, weighing 2–3 mg in total, were introduced into O2K chambers. Upon excision of BAT, a swift procedure was followed, whereby a small section, weighing approximately 2 mg, was rapidly excised. After adequate grinding, the resultant tissue suspension was collected and subsequently deposited into the O2K chamber for analysis. Maximal respiration flux was assessed using substrates including 5 mM pyruvate, 2 mM malate, 10 mM glutamate, and 2.5 mM adenosine diphosphate (ADP). Additionally, cytochrome c, at a concentration of 10 mM, was used to assess the integrity of the mitochondrial outer membrane. Rotenone (0.5 μM) was used to isolate respiration for analysis of complex I, 1 M succinate for complex II, and a solution of 2.5 μM antimycin A with for complex III.

### Sequence alignment and gene expression analysis

The BAT and muscle tissue samples from each group were collected for RNA-Seq. Total RNA was isolated using the TRIzol method (Thermo Fisher Scientific) following the manufacturer’s instructions. The quality of the RNA was assessed using the Agilent 2100 Bioanalyzer with the RNA 6000 Nano Kit from Agilent Technologies. cDNA libraries for each sample were constructed as previously described (60). The libraries were sequenced on the BGIseq500 platform (BGI-Shenzhen) using 150 bp paired-end reads with a target of 30 million reads per sample. The raw sequencing data were filtered using trim-galore (version 0.6.7) to remove reads containing sequencing adapters and low-quality bases. The resulting clean reads were aligned to the reference genome (mm10) using STAR (version 2.5.2b). Gene expression quantification was performed using RSEM (version 1.2.28) with commands rsem-calculate-expression --paired-end -p 20 –alignments samples.bam star_index.files. Differential gene expression analysis was conducted to compare MSTNΔUCP1 to MSTN^fl/fl^. DESeq2 (version 1.42.0) was used for differential expression analysis, considering genes with an adjusted *P* value of less than 0.05 and a fold change |FC| of greater than 1.5 as significant. Furthermore, KEGG pathway analysis was conducted using DEGs, while gene set enrichment analysis (GSEA) was performed using the obtained log_2_ FC values to detect pathways enriched with profiling genes.

### UPLC-MS/MS lipid profiling

Serum samples were thawed using an ice bath to minimize sample degradation. For lipid extraction, 10 μL serum was dispensed into individual wells of a 96-well plate. Subsequently, 300 μL extraction solution was introduced into each well. The plate underwent vortexing for a duration of 20 minutes, followed by centrifugation at 4,000*g* for 20 minutes (Allegra X-15R, Beckman Coulter). Subsequent to centrifugation, 20 μL supernatant was carefully transferred to a fresh 96-well plate and combined with 80 μL dilution solution. The resultant mixture was securely sealed, and the plate was prepared for the ultra-performance liquid chromatography coupled to tandem mass spectrometry (UPLC-MS/MS) analysis. The UPLC-MS/MS system was used for the precise quantification of all targeted metabolites.

Lipid profiling was conducted using the Waters ACQUITY UPLC system in conjunction with a Waters XEVO TQ-S MS, controlled by MassLynx 4.1 software (Waters). Chromatographic separation was achieved utilizing an ACQUITY BEH C18 column (1.7 μm, 100 mm by 2.1 mm internal dimensions, Waters). The gradient elution commenced at a flow rate of 0.4 mL/min, with an injected volume of 5 μL and the column temperature set at 40°C. Both mobile phases A and B comprised 5 mM ammonium formate in a solution of A (acetonitrile/H_2_O [95:5, v/v]) and B (acetonitrile/isopropanol [10:90, v/v]), respectively.

The elution of lipids followed the specified gradients: 0–2 minutes, 60% B; 2–8 minutes, 60%–100% B; 8–10 minutes, 100% B; and 10–12 minutes, 60% B. In the ESI+ mode, the capillary voltage was set at 3.2 kV. The desolvation gas flow was maintained at 1,000 liters/hour. The electrospray ion source temperature and desolvation temperature were held at 150°C and 500°C, respectively. Selected lipids were detected using the multiple reaction monitoring mode with the specified precursor and product ions, as previously described (61). The integration and quantification of raw lipid data generated by UPLC triple-quadrupole mass spectrometry were executed using the TargetLynx application manager (version 4.1, Waters).

### Cell lines, culture conditions, and transfection

Human HEK293T cells and murine macrophages were cultured in DMEM/H (Gibco, Thermo Fisher Scientific) with 10% FBS (Gibco, Thermo Fisher Scientific), 2 mM l-glutamine (Gibco, Thermo Fisher Scientific), and 1% penicillin-streptomycin (P/S) (Gibco, Thermo Fisher Scientific) in a 5% CO_2_ atmosphere at 37°C. Polyethylenimine (23966, PolySciences) was used for transfection according to the manufacturer’s instructions.

### Isolation and differentiation of primary brown adipocytes

Interscapular BAT was dissected from 10- to 14-day-old male mice and then minced and digested for 30 minutes at 37°C in isolation buffer (PBS containing 4% fatty acid–free BSA and 10 mg/mL collagenase D (Roche). Digested tissue was filtered through a 100 μm cell strainer to remove large pieces, and the flow-through was then centrifuged for 10 minutes at 600*g* to collect the supernatant (mature adipocytes) and the sediment (SVF cells). Then, the sediment resuspended cells and transferred to cell culture dishes. Differentiation of brown adipocytes was determined as previously described (62). To determine the effect of SMAD2/3 and the p38 signaling pathway, 10 μM p38 inhibitor, 20 nM SMAD2/3 inhibitor, 1 μM p38 agonist (MedChemExpress, SB202190, dehydrocorydaline or TP0427736 HCl), or 20 nM rMSTN (ACMEC biochemical, AC13218) was used to treat primary brown adipocytes.

### Coculture assay

To simulate the in vivo environment of obese mice, macrophages was induced inflammation by 200 nM palmitic acid (MilliporeSigma) (63). Macrophages were first induced with palmitic acid for 24 hours and then cocultured with mature adipocytes or BAT from Flox or BKO mice with or without rFGF21 protein for 24 hours. Finally, the cells were harvested for the experiments. The concentration of rFGF21 protein (MedChemExpress [MCE]) was used as previously described (26).

### Western blot analysis

Proteins were extracted with RIPA buffer (Beyotime Biotechnology) containing protease and phosphatase inhibitors (Beyotime Biotechnology) and were quantified using the Rapid BCA Protein Assay Kit (Thermo Fisher Scientific) according to the manufacturer’s instructions. Western blot analysis was performed as previous described (64). Briefly, 30 μg lysate was loaded onto SDS-PAGE gels, blotted onto PVDF membranes (MilliporeSigma), and incubated with antibodies. The primary antibodies used in this work included anti-MSTN (AF788, R&D Systems), anti-KLF4 (A13673, ABclonal), anti-PGC1α (NBP1-04676, Novus), anti-UCP1 (ab10983, Abcam), anti-LC3B (L7543, MilliporeSigma), anti-p62 (18420-1-AP, Proteintech), anti-PINK1 (sc-517353, Santa Cruz Biotechnology), anti-ATP5A (ab14748, Abcam), anti-ATP5A (ab14748, Abcam), anti-SDHB (10620-1-AP, Proteintech), anti-NDUFB8 (14794-1-AP, Proteintech), anti-p62 (18420-1-AP, Proteintech), anti–phosphorylated SMAD2/3 (anti–p-SMAD2/3) (8828S, Cell Signaling Technology [CST]), anti-SMAD2/3 (8685S, CST), anti–p-p38 (9211S, CST), anti-p38 (9212S, CST), anti–p-ERK (9101S, CST), anti-ERK (9102S, CST), anti–p-JNK (9251S, CST), anti-JNK (9252S, CST), anti-FGF21 (A23463, ABclonal), anti–β-tubulin (AC008, ABclonal), and anti-GAPDH (AB0037, Abways).

### Analysis of gene expression by qPCR

The TRIzol method (Thermo Fisher Scientific) was used to extract total RNA from tissues and cells according to the manufacturer’s instructions. Briefly, 1 μg RNA was converted into cDNA using the High-Capacity cDNA Reverse Transcription Kit (Thermo Fisher Scientific), and qPCR was performed with a QuantStudio 7 Flex Real-Time PCR System (Thermo Fisher Scientific) using SYBR Green PCR Master Mix (Accurate Biotechnology) according to the manufacturer’s instructions. Tbp, 36b4, or β-actin was used as the endogenous control. The primers used are listed in Table 1.

### Mitochondrial DNA copy numbers

Mitochondrial DNA (mtDNA) copy numbers were determined via quantitative qPCR as previously described (6). Briefly, total DNA was isolated from BAT using the Mouse Direct PCR Kit (B40015, Bimake) according to the manufacturer’s instructions. The mtDNA copy number was calculated from the ratio of COX II (mitochondria-encoded gene) to cyclophilin A (nucleus-encoded gene).

### ChIP-qPCR assay

Using the JASPAR website (https://jaspar.elixir.no/), we predicted there were 8 potential KLF4-binding elements on the Pink1 promoter. We performed the ChIP assay using the Sonication Chip Kit (ABclonal) according to the manufacturer’s instructions. In brief, we fixed 1 × 10^6^ primary brown adipocytes in 1% formaldehyde for 10 minutes at ambient temperature. The fixed cells were harvested, lysed, and sonicated for 35 cycles of 20 seconds ON/30 seconds OFF and 30% AMPL using SONICS VCX130 (SONICS). Antibodies against KLF4 (Proteintech, 11880-1-AP) and rabbit IgG (ABclonal) were used for immunoprecipitation. PCR amplification of the precipitated DNA was performed. The primer sequences used for the ChIP assay are listed in Table 1.

### Plasmid construction and lentivirus infection

The sequence encoding the shNA targeting MSTN was constructed by inserting a shRNA sequence (forward: CCGGCCTTTGGATGGGACTGGATTACTCGAGTAATCCAGTCCCATCCAAAGGTTTTTG; reverse: AATTCAAAAACCTTTGGATGGGACTGGATTACTCGAGTAATCCAGTCCCATCCAAAGG) into the AgeI/EcoRI site of pLKO.1-puro (Addgene). And packaging constructed shRNA of MSTN into the lentivirus. When the primary brown adipocytes reached 60% confluence, they were infected with lentivirus to knock down MSTN. The pCDNA3.1-KLF4 was constructed by amplifying PCR products from C57BL/6J mouse cDNA and inserting them into the EcoRI/XbaI site of pcDNA3.1 (OBiO Technology).

### AAV-KLF4 production and injection

pAAV-CMV-KLF4-3xFLAG-EF1-mNeonGreen-tWPA, pAAV-CMV-FGF21-3xFLAG-EF1-GdGreen-WPRE, and pAAV-U6-spgRNA (Mstn)-CMV-EGFP-WPRE was produced and purified by OBiO Technology. For the in vivo KLF4 and FGF21 expression experiment, a dose of 1.3 × 10^11^ genomic copies (GC) of AAV-KLF4 was in situ–injected into the interscapular BAT of BKO mice, and the same dose of AAV-GFP was in situ–injected into the interscapular BAT of Flox mice on an 8-week HFD. GTTs and ITTs were performed after 3 and 4 weeks of virus injection, respectively. For the in vivo MSTN-KO experiment, a dose of 1.3 × 10^11^ GC of AAV8-sgMstn was in situ–injected into the interscapular BAT of UCP1-Cre Cas9 mice, and the same dose of AAV8-sgCon was in situ–injected into the interscapular BAT of Cas9 mice.

### Statistics

The correlation between the expression of MSTN in s.c. fat and the BMI and the homeostatic model assessment for insulin resistance (HOMA-IR) was calculated by Spearman’s rank correlation coefficient. A 2-tailed Student’s *t* test was performed for 2-group comparisons. One-way ANOVA followed by Bonferroni’s post test was performed for intergroup comparisons. All data are presented as the mean ± SEM. The lipid-profiling analyses were performed in the R environment. A partial least-squares discriminant analysis (PLS-DA) model was constructed using the R package mixOmics. A Student’s *t* test was used to analyze significance of the metabolites between the groups. A *P* value of less than 0.05 was considered significant. The heatmap was constructed using the R package Complex Heatmap.

### Study approval

All animal studies were approved by the IACUC of the Shanghai University of Sport (102772022DW020).

### Data and software availability

The RNA-Seq data reported in this study have been deposited in the Gene Expression Omnibus (GEO) database (GEO GSE249030). All bioinformatics software used in the study are publicly available. All remaining data that support the findings of this study are available in the main text or the supplemental materials. See the Supporting Data Values file for values underlying the data presented in each graph and as the mean in the figures.

## Author contributions

XK and TL designed the research experiments. HW, SG, JD, XYK, SZ, YF, MH, HZ, WW, HL, KX, and HG performed the experiments. HW, YC, and YF performed the bioinformatics and metabolomics analyses. TL, SG, and XK analyzed the data. XK wrote the manuscript. All authors reviewed and contributed to the manuscript.

## Supplementary Material

Supplemental data

Unedited blot and gel images

Supporting data values

## Figures and Tables

**Table 1:**
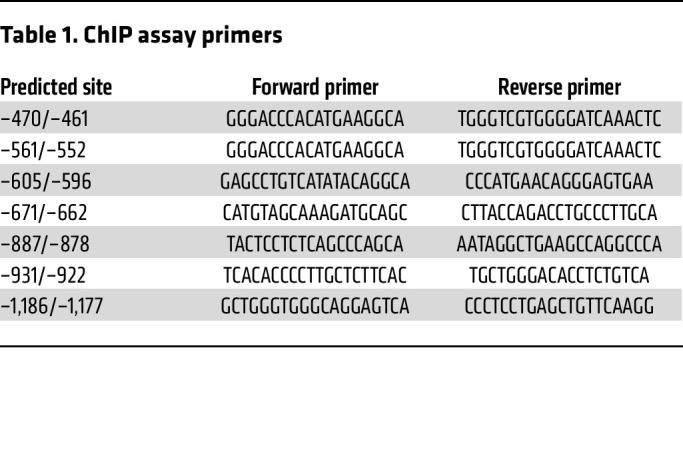
ChIP assay primers

## Version 1 06/18/2024

In-Press Preview

## Version 2 08/15/2024

Electronic publication
