# Supplementary material for: Myostatin regulates energy homeostasis through autocrine- and paracrine-mediated microenvironment communication
Source: J Clin Invest. 2024 Jun 18;134(16):e178303. doi: 10.1172/JCI178303 (PMC11324308; doi:10.1172/JCI178303)
Supplement: Supplemental data [file jci-134-178303-s150.pdf]

Extended Data Figure Legends

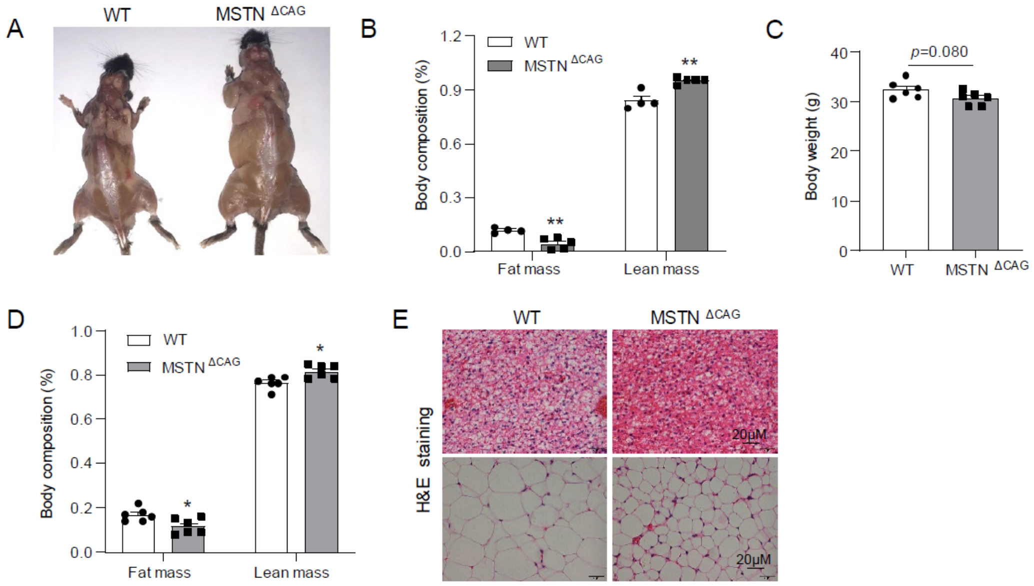

Extended Data Figure 1. (A) The morphology of male MSTN$^{\Delta CAG}$ and WT mice on chow diet. **(B)** The body composition of male MSTN$^{\Delta CAG}$ and WT (n= 4-5) on chow diet. **(C)** The body weight of male heterozygous of MSTN$^{\Delta CAG}$ and WT mice on 8-week HFD (n= 6). **(D)** The body composition of male heterozygous of MSTN$^{\Delta CAG}$ and WT mice on 8-week HFD (n= 6). **(E)** H&E staining of BAT, iWAT of male heterozygous of MSTN$^{\Delta CAG}$ and WT mice on 8-week HFD (scale bars, 20μm). All results were shown as mean ± SEM. $^{*}p<0.05$, $^{**}p<0.01$, compared with the WT group. A two-tailed Student t test was used for two groups statistical analysis.

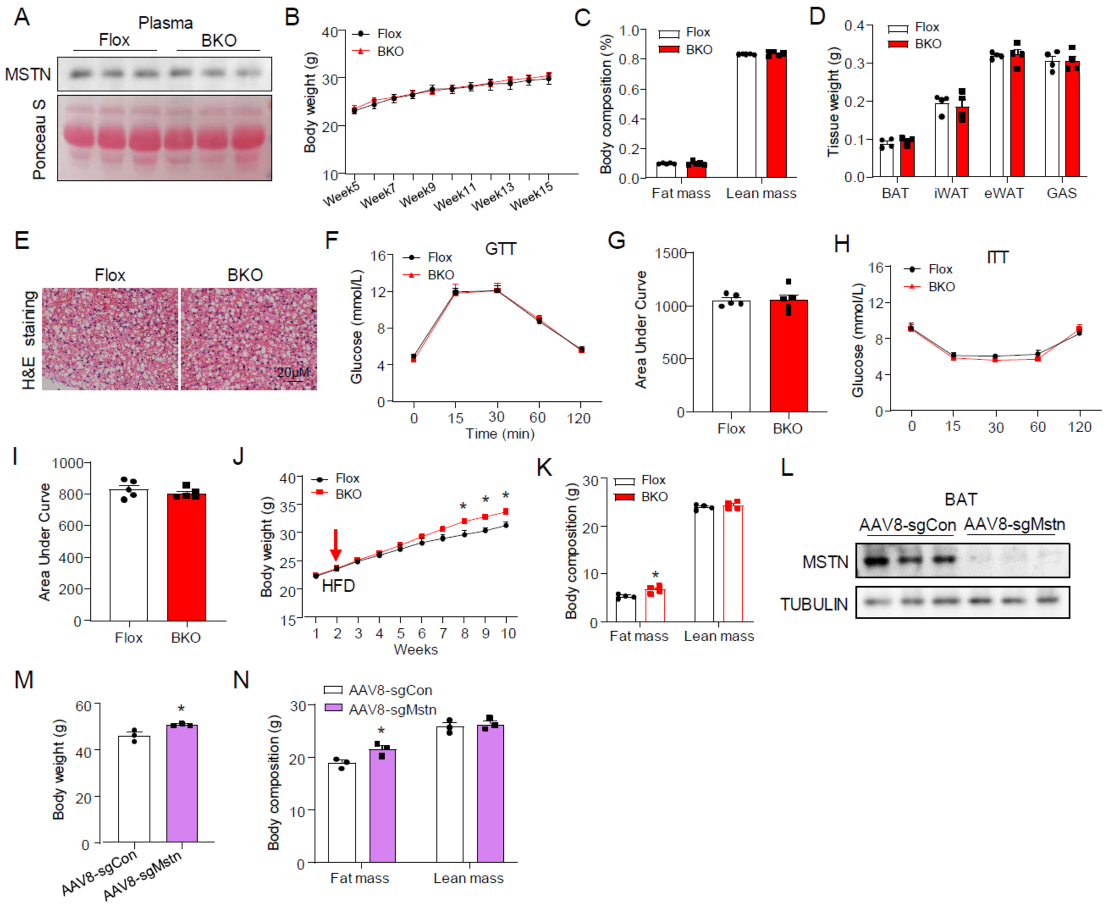

Extended Data Figure 2. (A) Western blot analysis of MSTN in plasma from BKO and Flox mice (n= 3). **(B)**The body weight of male BKO and Flox mice on chow diet (n= 5). **(C)** The body composition of male BKO and Flox mice on chow diet (n= 5). **(D)** The weight of BAT, iWAT, eWAT and GAS from male BKO and Flox mice on chow diet (n= 4). **(E)** H&E staining of BAT of male BKO and Flox mice on chow diet (scale bars, 20μm). **(F-I)** The glucose tolerance test (GTT) and insulin tolerance test (ITT) of male BKO and Flox mice on chow diet (n= 5). **(J)** The body weight of female BKO and Flox mice on HFD feeding (n= 4). **(K)** The body composition of female BKO and Flox mice on HFD feeding (n= 4). **(L)** Western blot analysis of MSTN in plasma from AAV8sgCon and AAV8sgMstn mice (n= 3). **(M)** The body weight of male AAV8sgCon and AAV8sgMstn mice (n= 3). **(N)** The body composition of male AAV8sgCon and AAV8sgMstn mice (n= 3). All results were shown as mean ± SEM. $^{*}p<0.05$, compared with the AAV8sgCon group. A two-tailed Student *t* test was used for two groups statistical analysis.

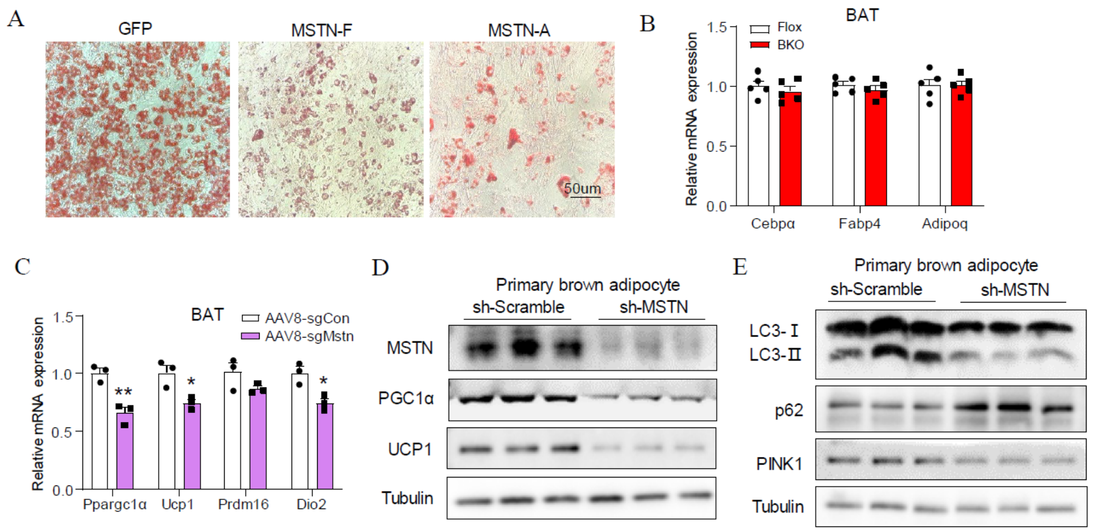

Extended Data Figure 3. (A) Oil red O staining of post-differentiated primary brown adipocytes (scale bar, 50μm). **(B)** Relative mRNA expression of adipogenic genes in BAT of Flox and BKO mice (n= 5). **(C)** Relative mRNA expression of thermogenesis genes in BAT of AAV8sgCon and AAV8sgMstn mice on HFD feeding (n= 3). **(D)** Western blot analysis of MSTN, PGC1-α and UCP1 in primary brown adipocytes with sh-Scramble and sh-MSTN groups (n= 3). **(E)** Western blot analysis of mitophagy proteins in primary brown adipocytes with sh-Scramble and sh-MSTN groups (n= 3). All results were shown as mean ± SEM. A two-tailed Student *t* test was used for two groups statistical analysis. MSTN-F, MSTN full-length, MSTN-A, the active MSTN.

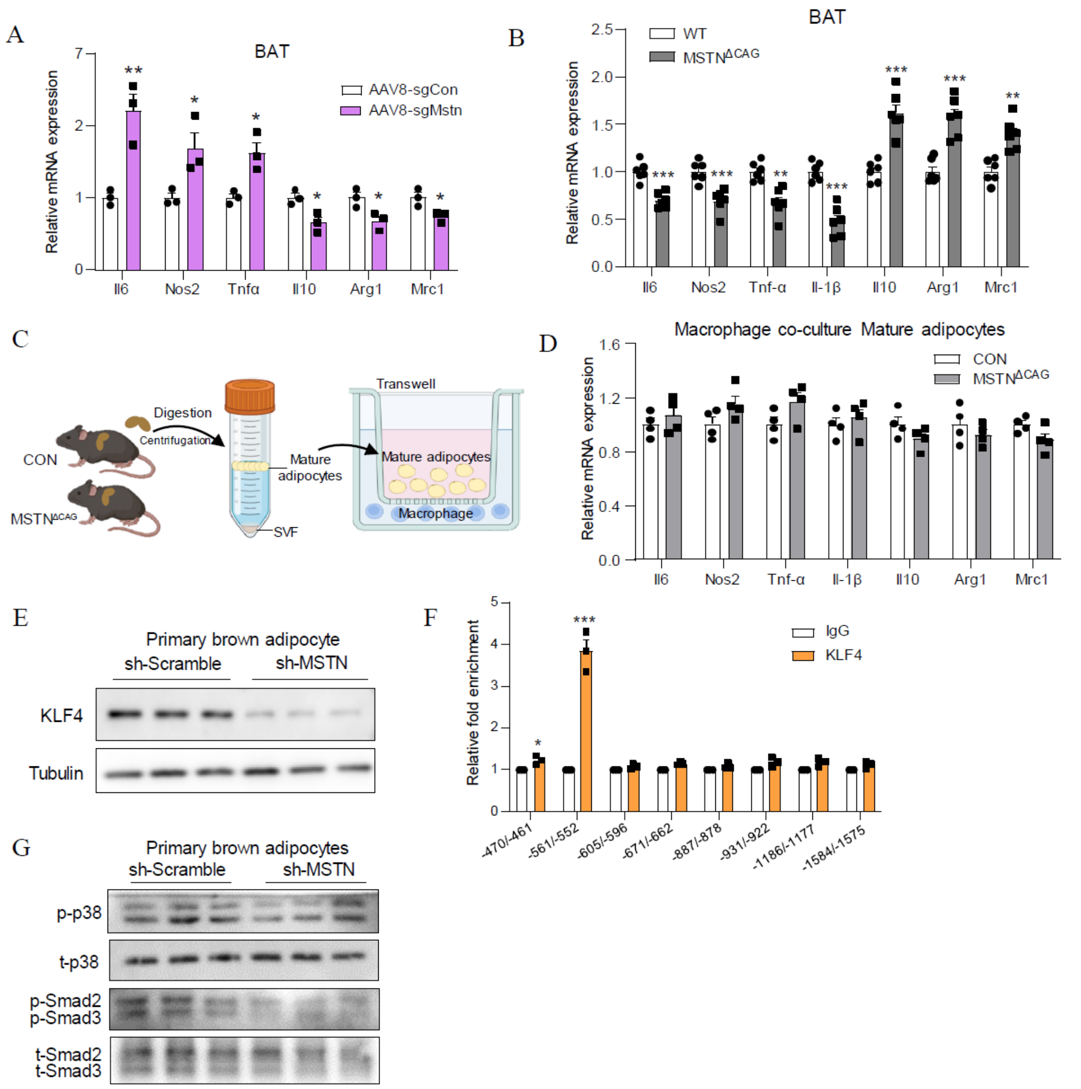

Extended Data Figure 4. (A) Relative mRNA expression of inflammatory genes in BAT of AAV8sgCon and AAV8sgMstn mice on HFD feeding (n= 3). **(B)** Relative mRNA expression of inflammatory genes in BAT of MSTN$^{\Delta CAG}$ and WT mice on chow diet (n= 6). **(C)** Schematic model of macrophage co-culture with mature adipocytes. **(D)** Relative mRNA expression of inflammatory genes in macrophage co-culture with mature adipocytes from MSTN$^{\Delta CAG}$ and CON mice (n= 4). **(E)** Western blot analysis of KLF4 in primary brown adipocytes with sh-Scramble or sh-MSTN (n= 3). **(F)** QPCR analysis of each ChIP-DNA sample was performed for Pink1, β-actin (n=3). Results are reported as fold enrichment of immunoprecipitated DNA from each sample relative to the DNA immunoprecipitated with the non-specific antibody, and were plotted in a scale in which the final value of IgG was arbitrarily set to 1. **(G)** Western blot analysis of the phosphorylation of Smad2/3 and p38 in primary brown adipocytes with sh-Scramble or sh-MSTN (n= 3). All results were shown as mean ± SEM. $^{**}p$<0.01, $^{***}p$<0.001, compared with the WT group. A two-tailed Student *t* test was used for two groups statistical analysis.

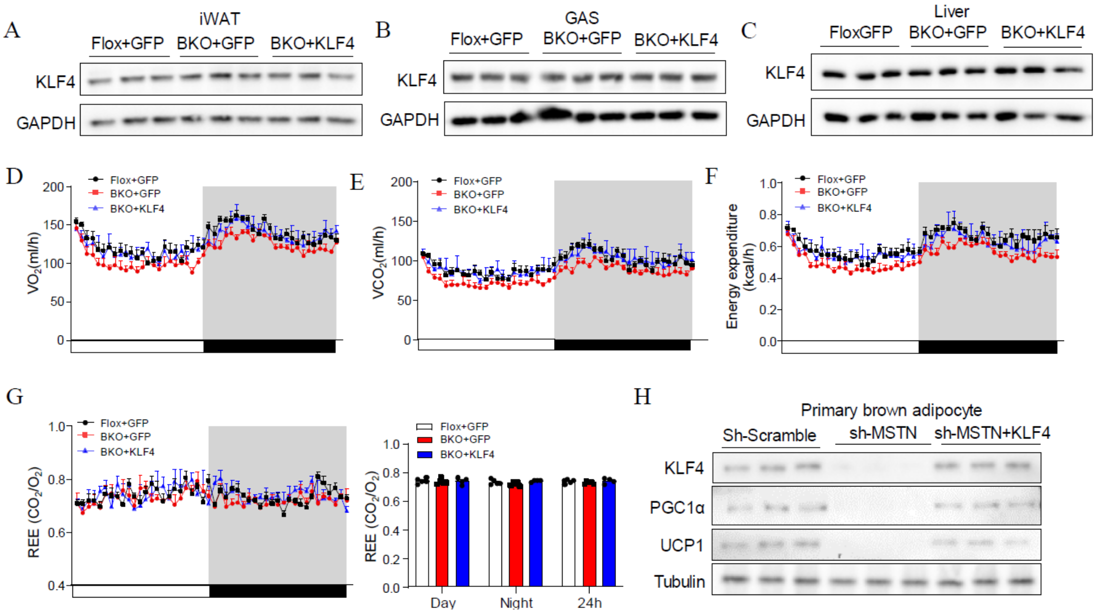

Extended Data Figure 5. (A-C) Western blot analysis of KLF4 in iWAT, GAS and Liver of Flox+GFP, BKO+GFP, and BKO+KLF4 mice on 12-week HFD (n= 3). **(D-F)** The oxygen consumption, carbon dioxide production, respiratory exchange rate and energy expenditure of male Flox+GFP, BKO+GFP, and BKO+KLF4 mice on 12-week HFD (n= 4-5). **(G)** Western blot analysis of KLF4, PGC1α and UCP1 in primary brown adipocytes with sh-Scramble, sh-MSTN or sh-MSTN+KLF4 (n= 3). All results were shown as mean ± SEM.

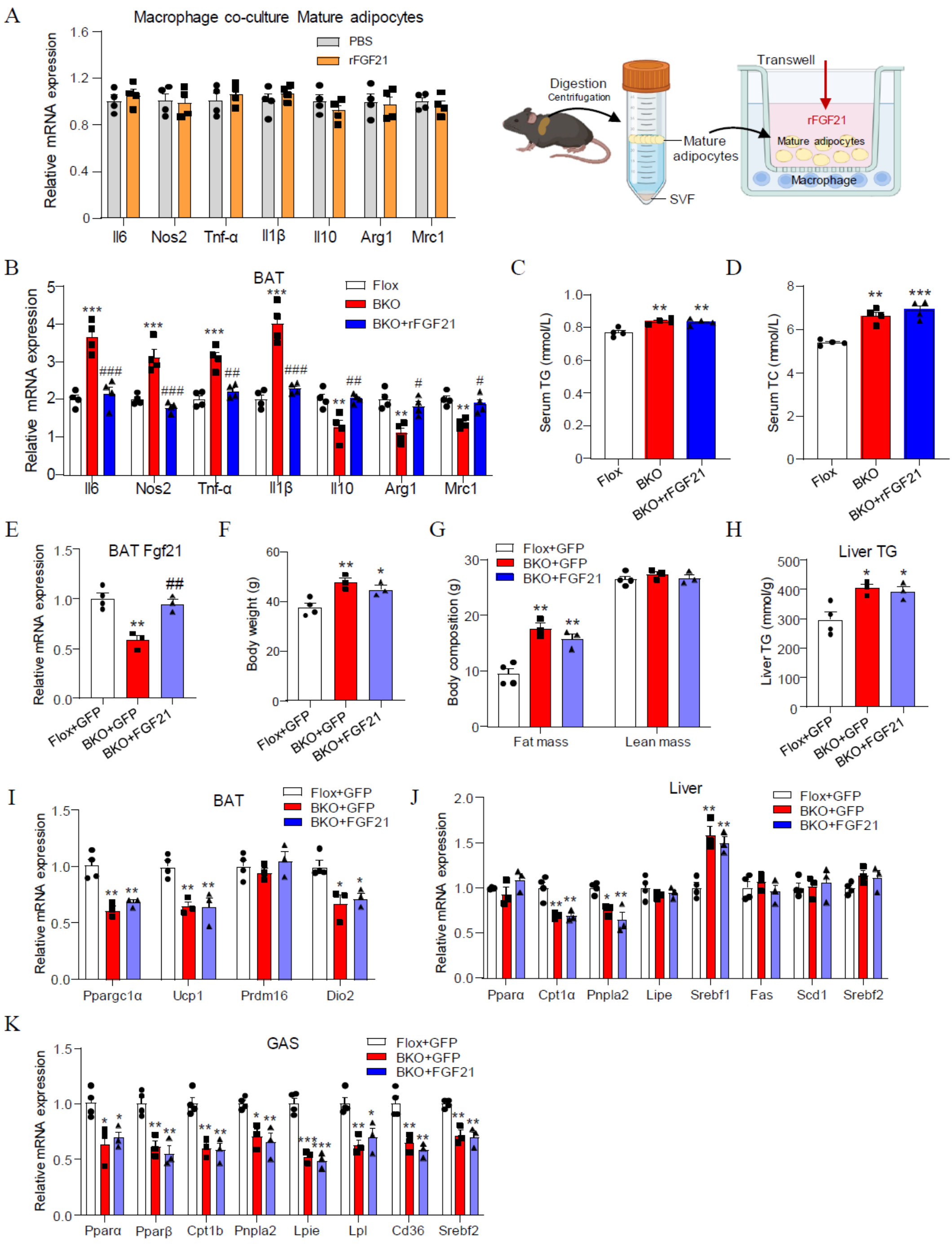

Extended Data Figure 6. (A) Relative mRNA expression of inflammatory genes in macrophage co-culture with mature adipocytes. rFGF21 group was additionally treated with 100 nM rFGF21 (n= 4). **(B)** Relative mRNA expression of inflammatory genes in BAT of Flox, BKO and BKO+rFGF21 mice (n= 4). **(C-D)** Plasma TG and TC levels in Flox, BKO and BKO+rFGF21 mice (n= 4). **(E)** Relative mRNA expression of Fgf21 in BAT of male Flox+GFP, BKO+GFP and BKO+FGF21 mice on HFD (n=3-4). **(F)** The body weight of male Flox+GFP, BKO+GFP and BKO+FGF21 mice on HFD (n=3-4). **(G)** Fat mass and lean mass in male Flox+GFP, BKO+GFP and BKO+FGF21 mice on HFD diet (n=3-

4). **(H)** The liver TG level in male Flox+GFP, BKO+GFP and BKO+FGF21 mice on HFD diet (n=3-4). **(I)** Relative mRNA expression of thermogenesis related genes in BAT of Flox+GFP, BKO+GFP and BKO+FGF21 mice on HFD (n= 3-4). **(J, K)** Relative mRNA expression of lipid metabolism related genes in liver and GAS of Flox+GFP, BKO+GFP and BKO+FGF21 mice on HFD (n= 3-4). All results were shown as mean ± SEM. $^{**}p<0.01$, $^{***}p<0.001$, compared with the Flox group. $^{\#}p<0.05$, $^{\#\#}p<0.01$, $^{\#\#\#}p<0.001$, compared with the BKO group. A one-way ANOVA followed by Bonferroni post-tests was used for three groups statistical analysis.
