# Supplementary material for: Myostatin regulates energy homeostasis through autocrine- and paracrine-mediated microenvironment communication
Source: J Clin Invest. 2024 Jun 18;134(16):e178303. doi: 10.1172/JCI178303 (PMC11324308; doi:10.1172/JCI178303)

Uncropped gels for Western Blots in Figure 1

F1-A

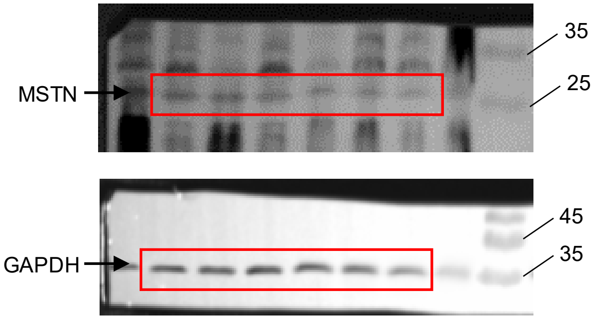

F1-C

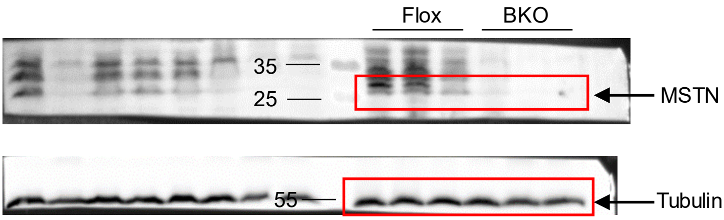

Uncropped gels for Western Blots in Figure 4

F4-B

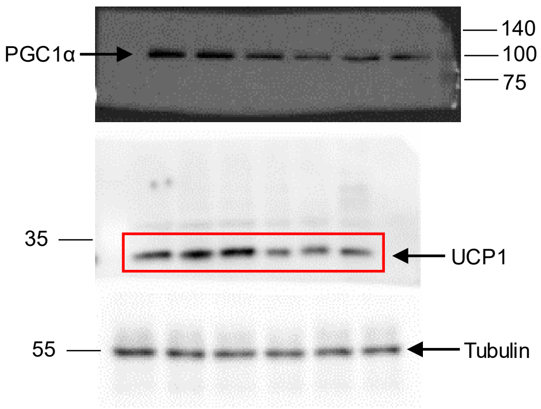

F4-F

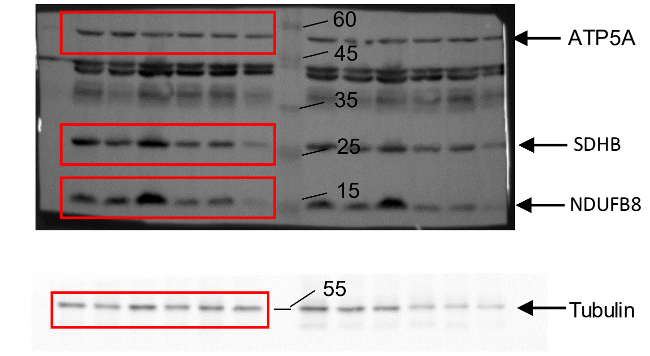

F4-G

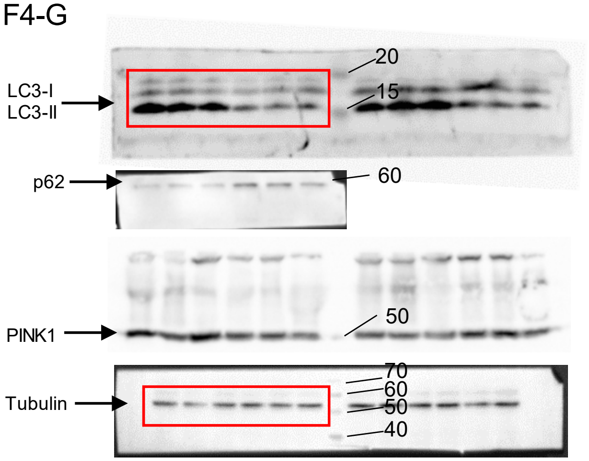

F4-M

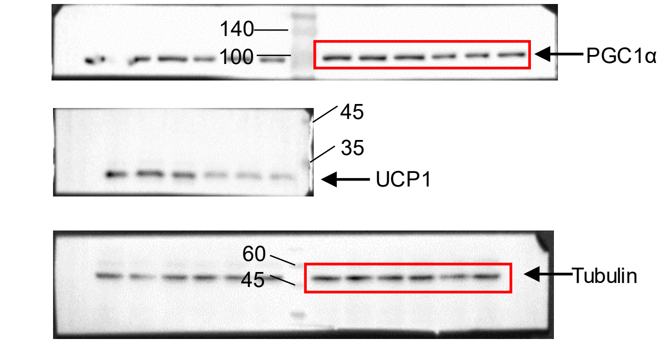

F4-O

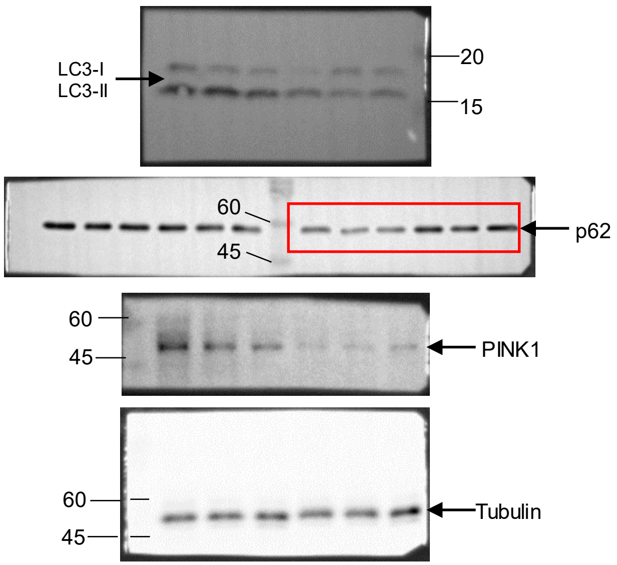

Uncropped gels for Western Blots in Figure 5

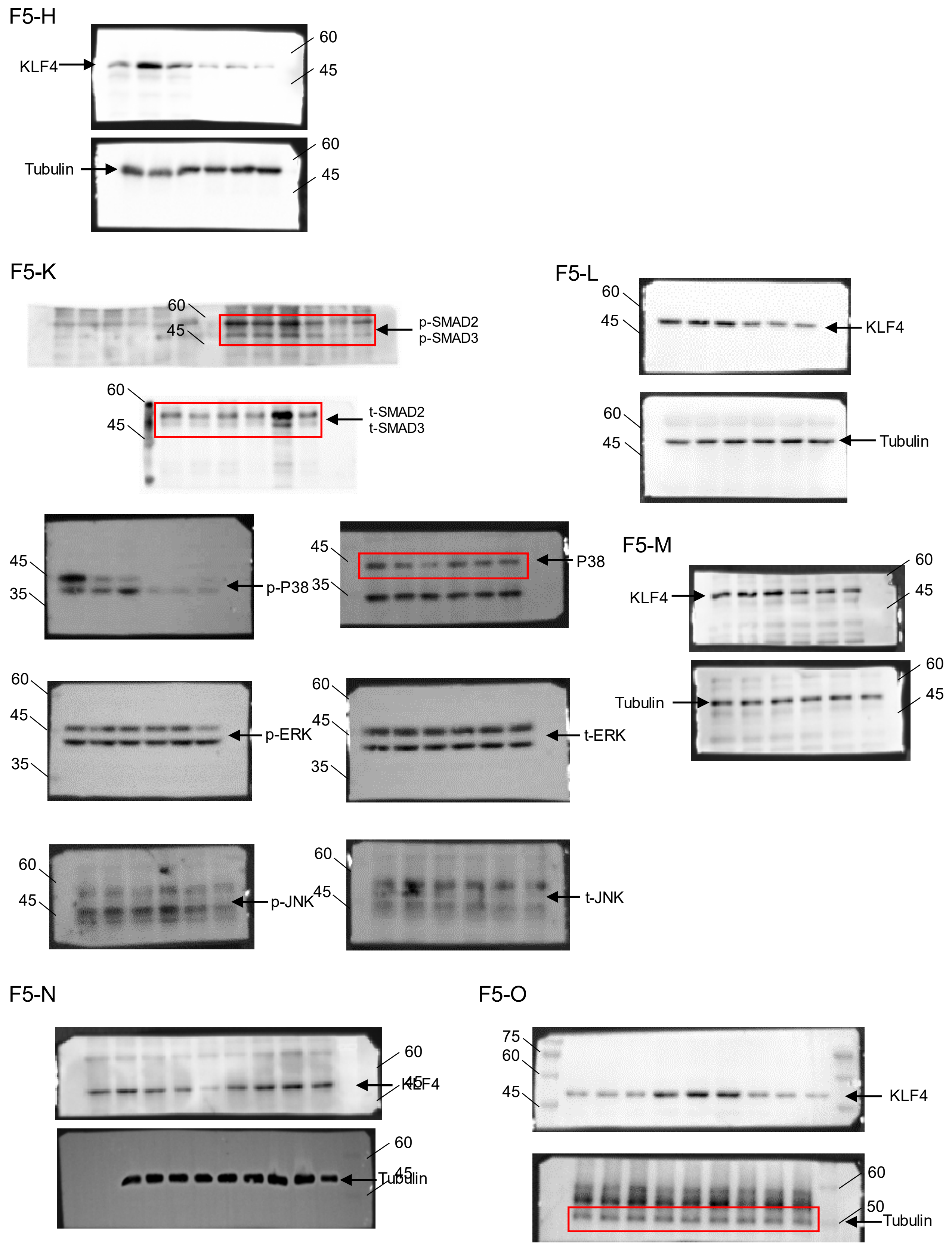

Uncropped gels for Western Blots in Figure 6

F6-A

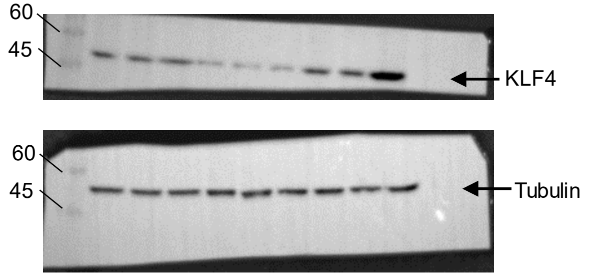

F6-P

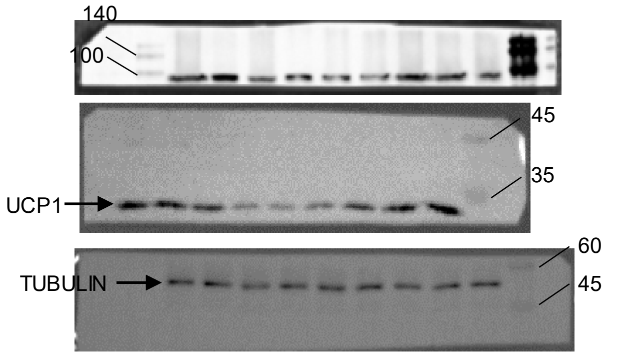

F6-S

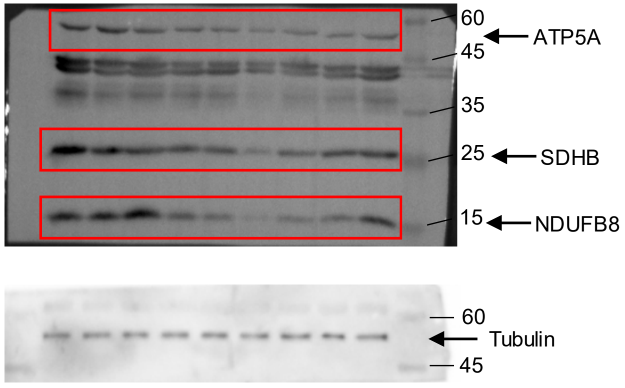

F6-T

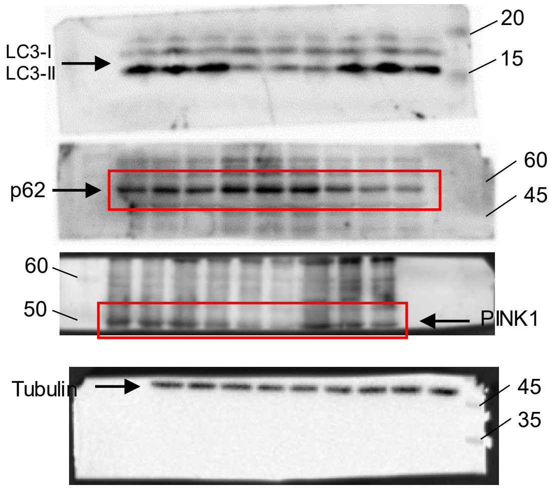

Uncropped gels for Western Blots in Figure 7

F7-A

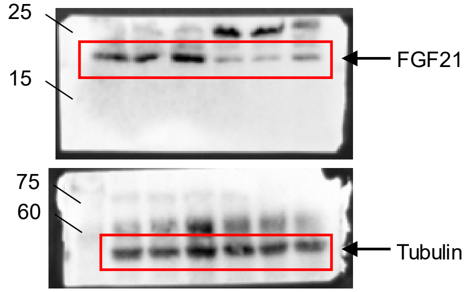

F7-D

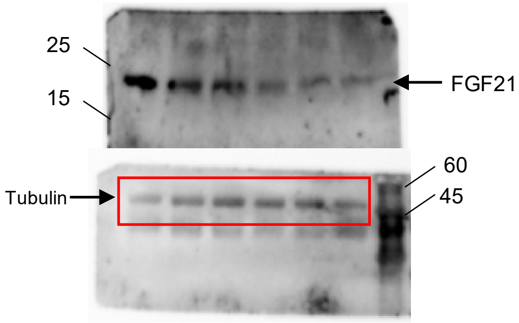

F7-E

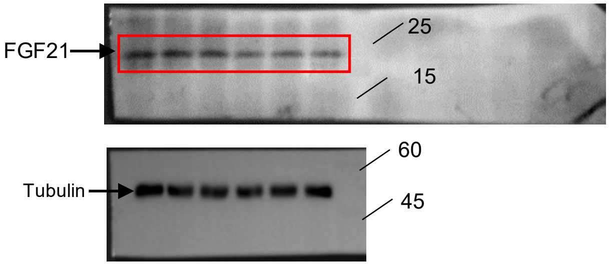

F7-F

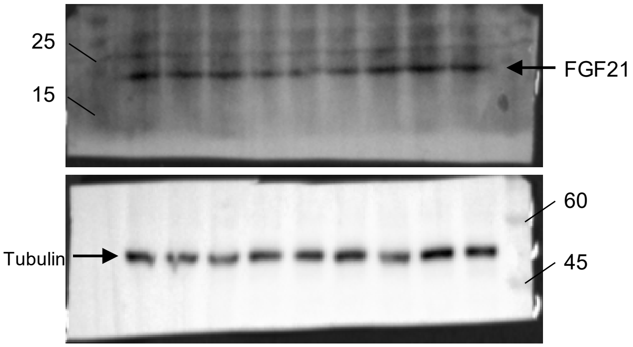

F7-G

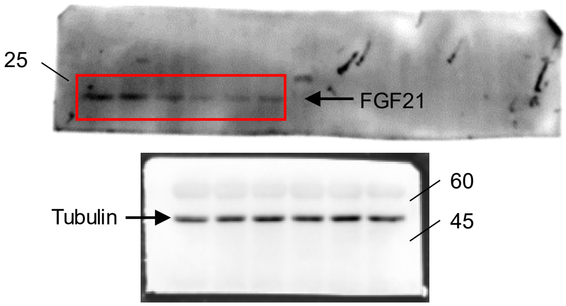

F7-H

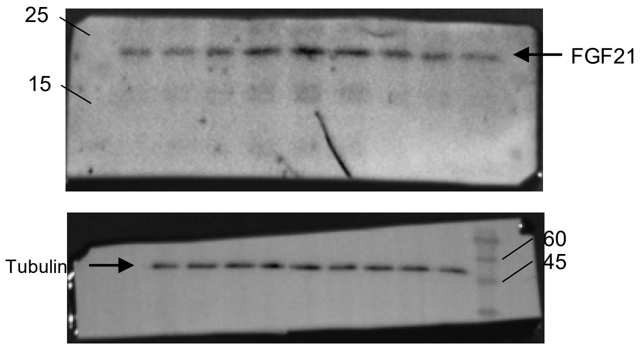

Uncropped gels for Western Blots in Extended Data Fig. 2

Extended Data Fig. 2A

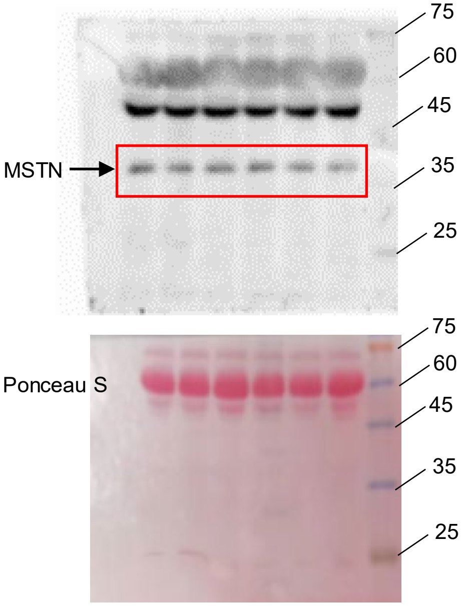

Extended Data Fig. 2L

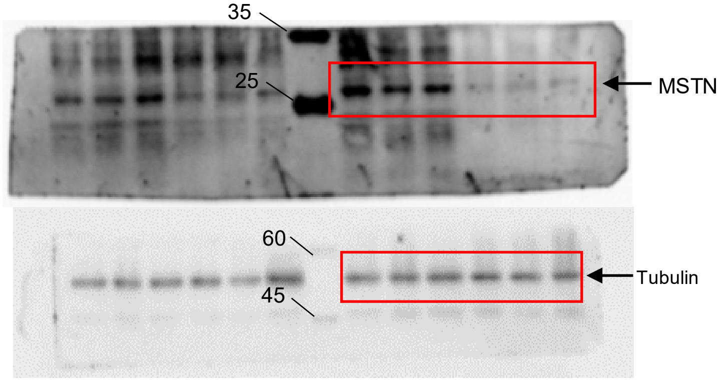

Uncropped gels for Western Blots in Extended Data Fig. 3

Extended Data Fig. 3D

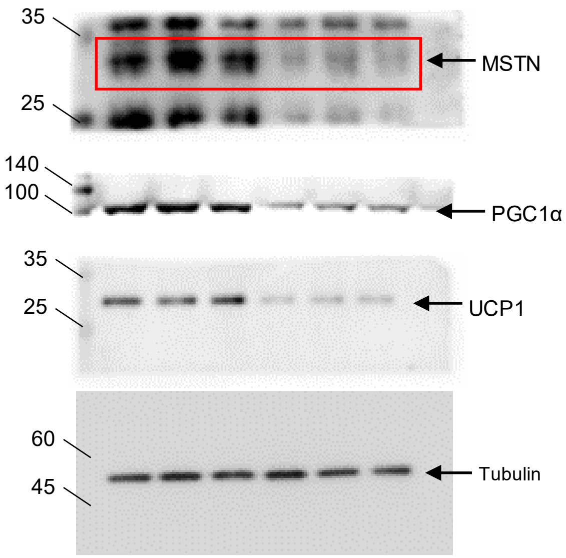

Extended Data Fig. 3E

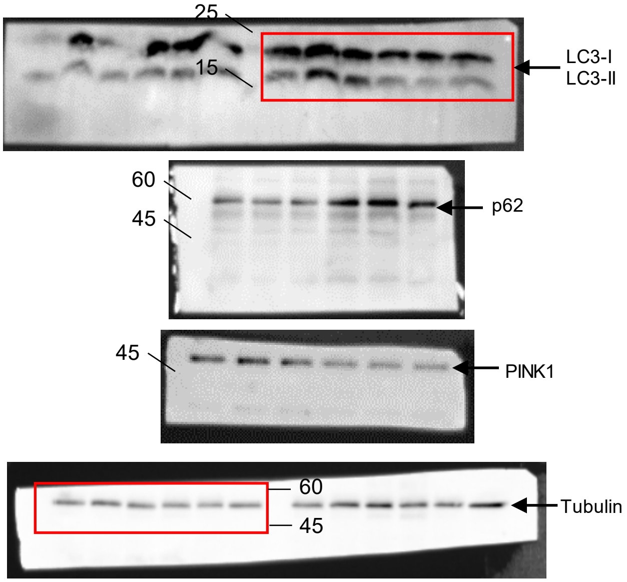

Uncropped gels for Western Blots in Extended Data Fig. 4

Extended Data Fig.4E

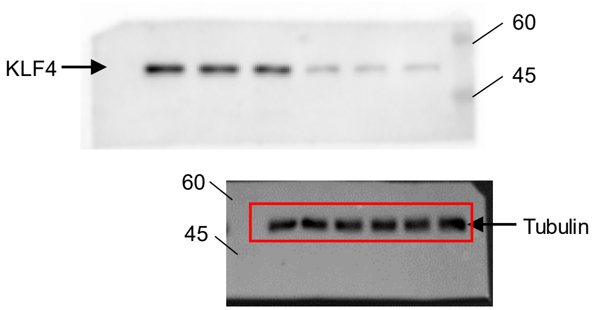

Extended Data Fig.4G

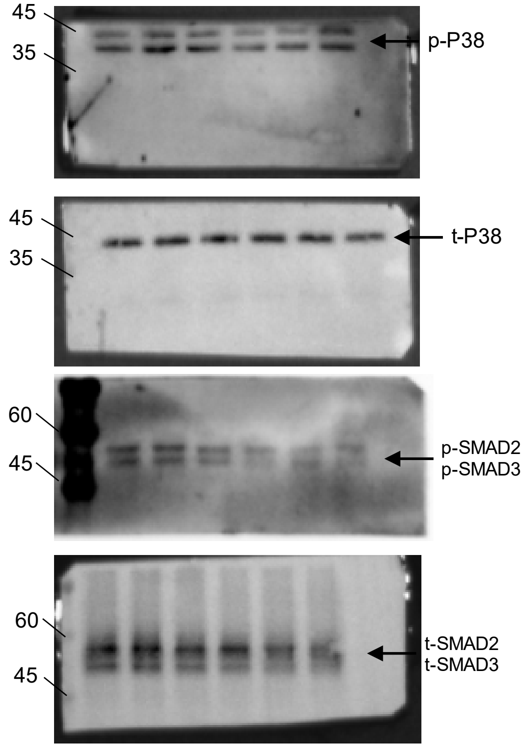

Uncropped gels for Western Blots in Extended Data Fig. 5

Extended Data Fig.5A

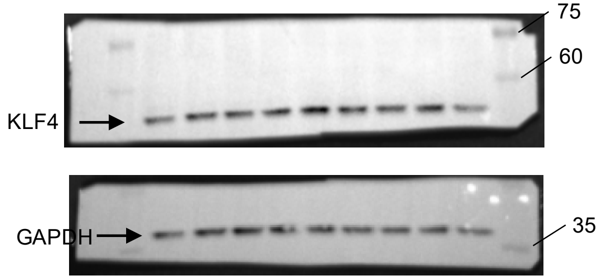

Extended Data Fig. 5B

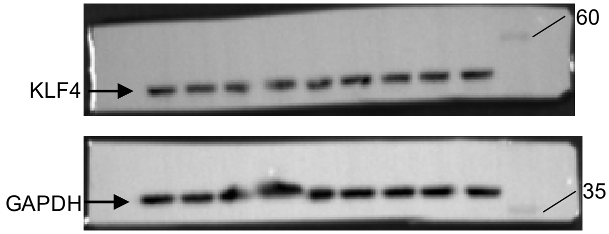

Extended Data Fig. 5C

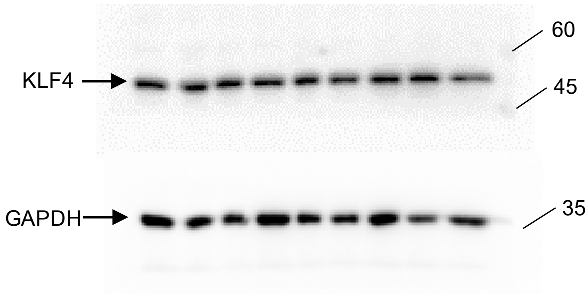

Extended Data Fig. 5H

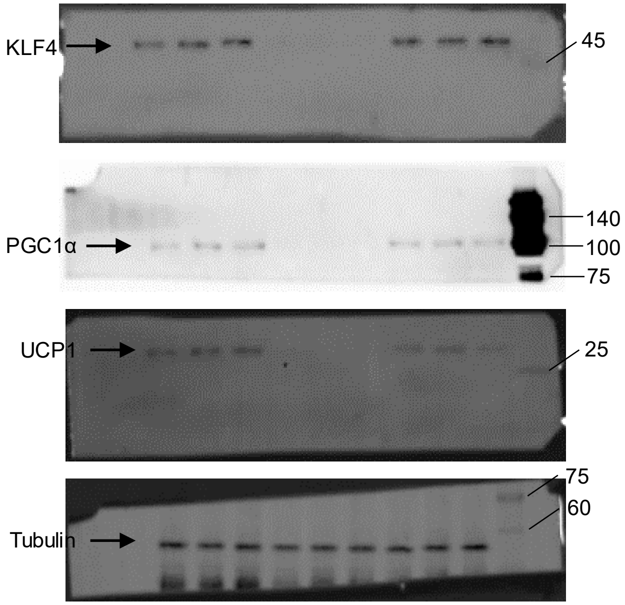

Supplement: Unedited blot and gel images [file jci-134-178303-s151.pdf]
